# Control of Fusarium wilt by wheat straw is associated with microbial network changes in watermelon rhizosphere

**DOI:** 10.1038/s41598-020-69623-6

**Published:** 2020-07-29

**Authors:** Lili Tang, Ye Xia, Chao Fan, Jinming Kou, Fengzhi Wu, Wenhui Li, Kai Pan

**Affiliations:** 1 College of Horticulture and Landscape Architecture, Northeast Agricultural University, 600 Changjiang Street, Harbin, 150030 Heilongjiang China; 2 Key Laboratory of Biology and Genetic Improvement of Horticultural Crops (Northeast Region), Ministry of Agriculture/Northeast, Agricultural University, Harbin, 150030 Heilongjiang China; 3 Institute of Cash Crops, Heilongjiang Academy of Agricultural Sciences, Harbin, 150086 Heilongjiang China; 4 Institute of Crop Cultivation and Tillage, Heilongjiang Academy of Agricultural Sciences, Harbin, 150086 Heilongjiang China; 5 Department of Plant Pathology, Ohio State University, Columbus, OH 43210 USA

**Keywords:** Microbial ecology, Microbiome

## Abstract

Straw return is an effective strategy to alleviate soil-borne diseases. Though watermelon Fusarium wilt is a severe soil-borne disease, the effect of wheat straw on the disease remains unclear. Thus, we investigated the effects of wheat straw on soil bacterial and fungal communities by adding wheat straw to consecutive watermelon soil in the greenhouse condition. The microbiome changes were further investigated using network analysis based on 16S rDNA and internal transcribed spacer deep sequencing. Wheat straw addition increased the fungal community diversity, whereas the bacterial diversity was not affected. Compared to the control group, the relative abundance of some bacteria, including *Actinobacteria*, *Chloroflexi*, and *Saccharibacteria*, was increased with wheat straw addition. For fungi, the relative abundance of *Fusarium* was decreased with wheat straw addition. Microbial network analysis demonstrated that the fungal community has a more complex connection than the bacterial community. In addition, redundancy analysis indicated that the *Fusarium* genera were significantly related to the disease index. Taken together, the addition of wheat straw might affect the microbial community through increasing the relative abundance of phylum *Actinobacteria*, decreasing the relative abundance of *Fusarium,* and increasing the fungal network complexity to enhance the defense of watermelon against Fusarium wilt disease.

## Introduction

Watermelon is the second most produced economic fruit widely cultivated all over the world. However, the economic benefits and planting convenience have led to the repetitive growth of watermelon plants on the same land, which has caused severe soil-borne diseases globally. For instance, long-term monoculture has led to the disease burst of watermelon Fusarium wilt^1^. As the most destructive soil-borne disease affecting watermelon yields worldwide, Fusarium wilt is caused by the fungi *Fusarium oxysporum* f. sp. *niveum* (FON). This disease causes approximately 30–50% of watermelon yield losses worldwide^2^.

Continuous cropping monoculture reduces soil microbial diversity, increases the accumulation of pathogens, and aggravates the occurrence of plant diseases^3^. Soil microorganisms are key factors for maintaining soil health and suppressing plant diseases^4^. The soil microbial community exists in complicated interaction systems^5^. In the soil, bacterium and fungi are the most prevalent and diverse microbial organisms^6^. Alteration of the soil microbial community structure had been identified as the key factor that leads to the accumulation of fungal pathogens under the continuous cropping monoculture system^3^. However, increased microbial diversity and rebalanced composition could reduce pathogen invasion and delay disease development by increasing nutrient competition, enhancing host immunity, and improving soil health^7^. Continuous cropping monoculture leads to an increase in pathogen abundance at the expense of plant benefits, increasing soil-borne diseases^8^. For instance, an adverse connection between soil-borne plant illnesses and soil microbiota diversity was identified by Mazzola et al.^9^. Penton et al*.*^10^ reported that the higher the fungal diversity was, the stronger the potential of disease suppression in the soil became. It was reported that watermelon intercropping with wheat could alleviate Fusarium wilt by altering the rhizosphere soil microbiomes^11^. Previous research also showed that wheat cover crops could promote cucumber growth by regulating soil microbial community diversity^12^.

Microbial network assessment is commonly utilized to reveal soil ecosystem microbial linkages^13^. Wu et al*.*^14^ employed network analysis to show that there were more connected relationships in the fungal communities in suppressive soil than in the soil of a consecutive monoculture system. Network analysis by Shen et al*.*^15^ also revealed that the number of years of continuous monoculture cropping changed the microbial dominance from bacteria to fungi and from cooperative to competitive interactions. Network analysis could therefore add substantial dimensions and provide insights into the combination of microorganisms for better understanding of the complex interactions among soil microbes, which is not only limited to the diversity and composition.

Fusarium wilt was previously controlled by the use of crop rotation^16^^,^ fumigation^17^^,^ fungicides^18^^,^ and microbial antagonists^19^. However, these measures are generally impractical due to the economic cost, time, labor consumption, and even environmental pollution. Recently, the development of biological control approaches and the discovery of novel natural pathogen antagonists have become research hotspots^11,12,20^. Crop straw is extensively used in modern agriculture worldwide, and is also the oldest and most economic management practice to relieve monoculture problems and increase crop yields and quality. In addition, crop straw incorporation can improve the physical and biological conditions of the soil and reduce the occurrence of soil-borne diseases^21^. And crop straw came from aboveground and there are no reports indicating that the microbes on crop straw can cause disease. For instance, adding crop straw led to changes in the compositions of the bacterial and archaea community in paddy soil^22^^,^ as well as the microbiota of rice rhizosphere^23^. Further, rice straw mulching reduced the sheath blight of wheat by increasing total bacterial and *Pseudomonas fluorescens* populations^24^. A large number of studies have shown that straw can inhibit the occurrence of crop diseases by altering the composition, proportion, and taxonomic structure of soil microbiota.

Our previous study investigated the effects of five straws, specifically wheat straw (X00, X01, and X03), rice straw, and maize straw on the inhibition of watermelon Fusarium wilt disease in the continuous watermelon monoculture system. We found that X01 resulted in the lowest disease incidence and severity compared to X00, X03, rice straw, and maize straw, without the addition of crop straw (CK) (see Supplementary Fig. S1). Therefore, X01 was selected as the experimental material for further analysis in this study. In the present work, we hypothesized that wheat straw (X01) could decrease the incidence and severity of watermelon Fusarium wilt disease by increasing plant-beneficial microorganisms and inhibiting the pathogen (FON) population, or changing the composition of watermelon rhizosphere bacterial and fungal communities. In order to test this hypothesis, the microbial community compositions of 36 soil samples from the continuous watermelon monoculture cropping system with and without wheat straw addition at both the flowering and fruiting stages were analyzed using Illumina MiSeq targeted amplicon sequencing. In addition, the microbial network composition and interactions constructed by the phylogenetic molecular ecological network (pMEN) were examined. Our study revealed that community factors such as changes in the structure and molecular ecological network could lead to the increased abundance of beneficial microbes and decreased abundance of harmful pathogens for watermelon Fusarium wilt disease control.

## Results

### Bacterial community composition

From 36 soil specimens, 3,370,643 high-quality 16S rDNA reads were obtained [(CK1, T1, CK2, and T2 treatments) × 9 replicates] with 74,955–103,931 sequencing reads (mean = 91,908) per sample. The maximum read length was 478 bp and the minimum average length was 341 bp for the 16S rDNA genes. All rarefaction curves for the bacterial samples revealed that the amount of recorded OTUs was generally 7,000 reads per plateau, indicating the assessment adequately covered the microbial variety (see Supplementary Fig. S2a). The bacterium richness (Chao1 and ACE), evenness indexes (Shannon and Simpson), and number of OTUs between CK1 and T1 as well as between CK2 and T2 were not significantly different (see Supplementary Table S1a).

**Figure 1:**
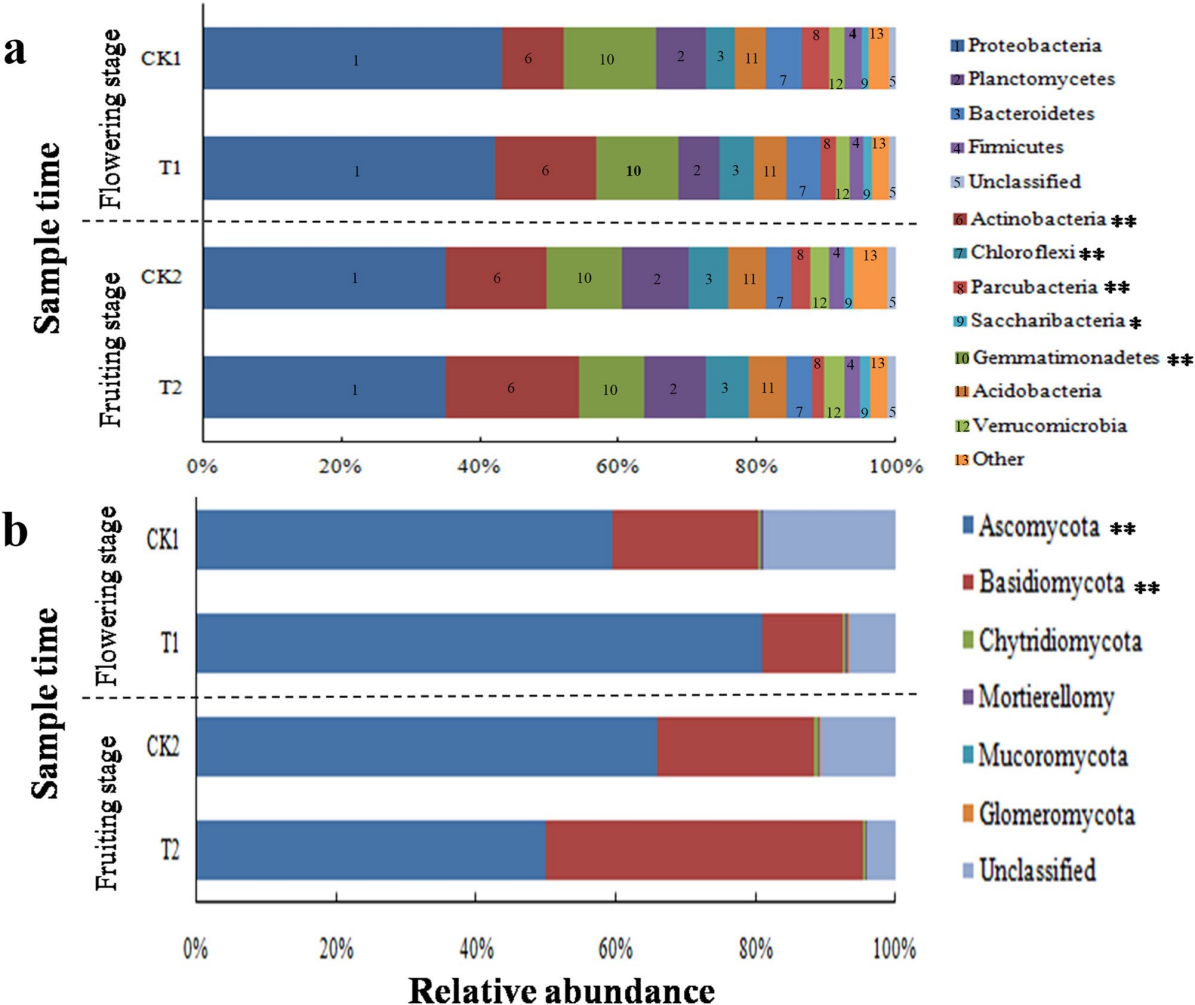
Major bacterial (**a**) and fungal phylum (**b**) relative abundance in the soil with (T1 and T2) and without (CK1 and CK2) wheat straw addition. Bacterial phyla with > 1% and fungal phyla with > 0.1% average relative abundances. Others included bacterial phyla below 1% relative abundance and unidentified bacterial and fungal phyla. According to the Student's t-test (n = 9), * and ** represented *P* < 0.05 and *P* < 0.01 between soil with (T1 and T2) and without (CK1 and CK2) wheat straw addition, respectively.

The soil bacterial composition of the two treatment groups at the two growth periods were compared at the level of the phylum. A total of 26 bacterial phyla were identified, with the exception of 1.03% of unclassified sequencing reads. The main phyla of the sequenced bacteria were *Proteobacteria*, *Actinobacteria*, and *Gemmatimonadetes*, which occupied over 64.5% of the total bacterial populations in the sample sequences. *Chloroflexi*, *Acidobacteria*, *Bacteroidetes*, *Parcubacteria*, *Verrucomicrobia*, and *Firmicutes* were also identified at relatively elevated richness (average relative abundance > 1%) (Fig. 1a). Wheat straw addition significantly increased the relative abundances of *Actinobacteria*, *Chloroflexi*, and *Saccharibacteria*, while significantly decreasing the relative abundance of *Parcubacteria* at both moments of sampling (*P* < 0.05) in the consecutive watermelon monoculture system. The abundance of *Gemmatimonadetes* was visibly more elevated in T1 than in CK1 and no significant differences between T2 and CK2 (*P* > 0.05) were detected (see Fig. 1a and Supplementary Table S2a).

### Fungal community composition

For fungi, 4,991,373 high-quality fungi ITS gene sequence reads were derived from the 36 soil samples tested with 89,803–194,007 sequence reads (mean = 138,649) per sample. The maximum read length was 499 bp and the minimum average length was 201 bp for the ITS genes. At a 97% sequence similarity for all samples, the OTU rarefaction curves appeared to reach the saturation plateau (see Supplementary Fig. S2b), indicating the assessment has covered the microbial variety. The fungal ACE and Chao1 indexes were significantly higher in T1 than in CK1, whereas the differences were not significant between CK2 and T2. Moreover, the fungal Shannon index was significantly higher in T2 than in CK2, and no significant differences were detected between T1 and CK1. The two treatments and two watermelon growth stages did not differ in the amount of OTUs (see Supplementary Table S1b).

In all soil specimens, *Ascomycota* and *Basidiomycota* were the two prevalent fungal phyla, accounting for over 89% of the total reads. Wheat straw addition significantly enhanced the relative richness of *Ascomycota* while considerably reducing the relative abundance of *Basidiomycota* during the flowering stage in the consecutive watermelon monoculture system. On the contrary, the relative abundance of *Ascomycota* was greater without wheat straw compared to that with wheat straw, whereas the relative abundance of *Basidiomycota* was lower without wheat straw compared to that with wheat straw in the fruiting stage. The relative abundance of *Chytridiomycota*, *Mortierellomy*, *Mucoromycota*, and *Glomeromycota* was not significantly different with and without wheat straw (see Supplementary Fig. 1b and Table S2b).

### Taxonomic characteristics of bacterial and fungal communities

**Figure 2:**
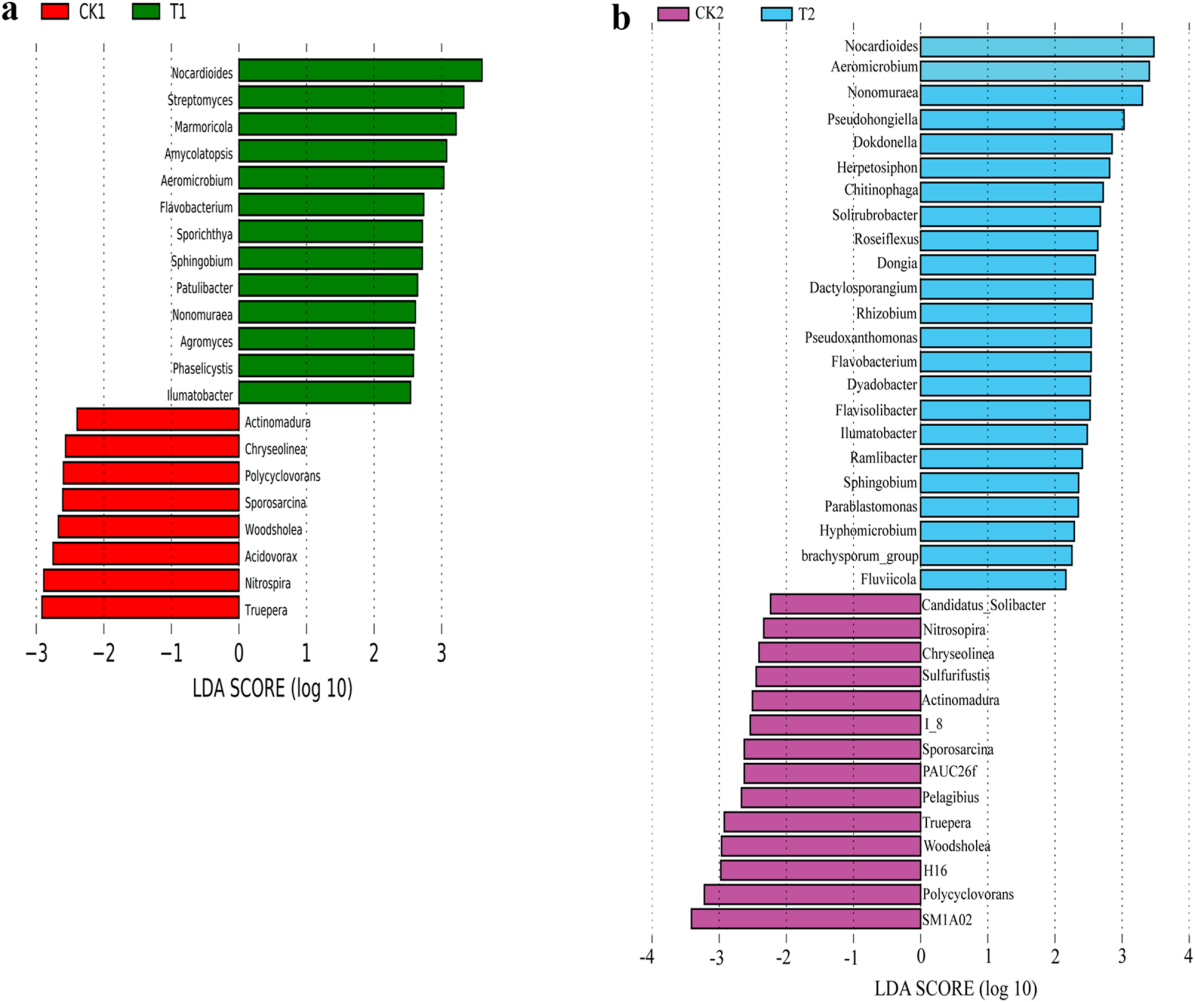
LDA histogram scores for bacterial genera with different abundance for the flowering stage (**a**) and fruiting stage (**b**) in the watermelon monoculture system.

LEfSe was further used to assess the association of the bacterial genera (average relative abundances > 0.1%) in the microbial community in the soil with (T1 and T2) and without (CK1 and CK2) wheat straw addition in the flowering and fruiting stages. The bacterial genera, *Sporosarcina*, *Nitrospira*, *Truepera*, *Acidovorax*, *Woodsholea*, *Polycyclovorans*, *Actinomadura*, and *Chryseolinea* were more abundant in CK1 than in T1 (Fig. 2a). *Sphingobium*, *Phaselicystis*, *Flavobacterium,* and some *Actinobacteria* (*Sporichthya*, *Aeromicrobium*, *Nonomoricola*, *Marmoricola*, *Ilumatobacter*, *Streptomyces*, *Agromyces*, *Patulibacter*, *Amycolatopsis,* and *Nocardioides*) were more abundant in T1 than in CK1 (Fig. 2a). *Actinobacteria* was overdominant in T1, taking up 4.30% of the relative abundance with > 0.1% bacterial reads in T1 soil (only 0.04% in CK1 soil) (see Supplementary Table S3a). In addition, 14 bacterial genera with relative abundance > 0.1% were more prevalent in CK2 samples, including *Spororosarcina*, *Chryseollinea*, *Nitrososporia*, *Truepera*, *Actinomadura*, two *Planctomycetes* (SM1A02 and I-8), and some *Ptoteobacteria* (*Sulfurifustis*, *Polycyclovorans*, *Woodsholes,* and *H16*) (Fig. 2b). In contrast, the *Actinobacteria* (*Aeromicrobium*, *Nonomured*, *Nocardioides*, *Dactylosporangium,* and *Ilumatobacter*) group and *Proteobacetia* (*brachysporum_group*, *Ramlibacter*, *Dongia*, *Hyphomicrobium*, *Rhizobium*, *Sphingobium*, *Parablastomonas*, *Pseudoxanthononas*, *Dokdonella,* and *Pseudohoniella*) group were more abundant in the T2 soil than in CK2 (see Fig. 2b and Supplementary Table S3b).

**Figure 3:**
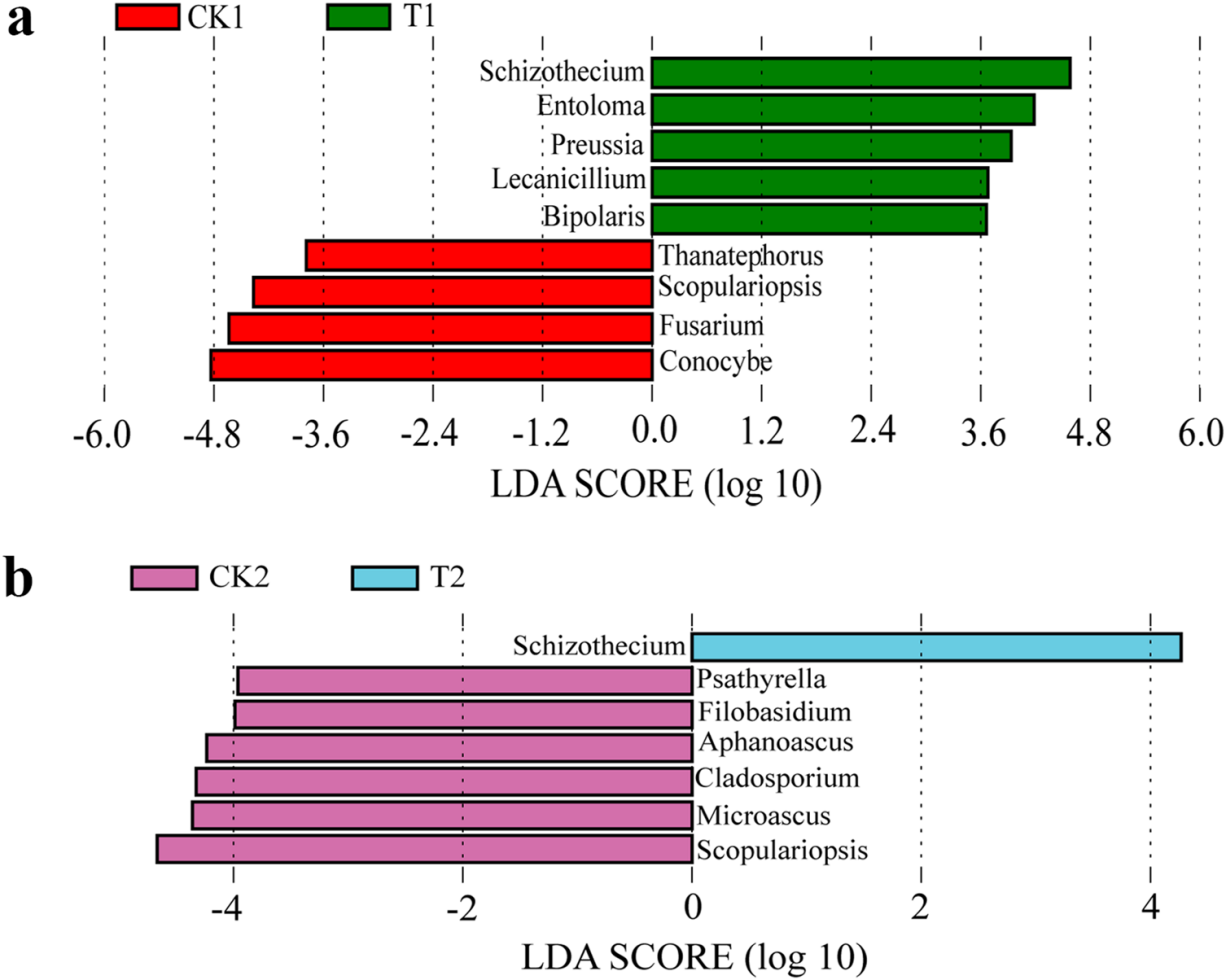
LDA histogram scores for fungal genera with different abundance for the flowering stage (**a**) and fruiting stage (**b**) in the watermelon monoculture system.

With respect to the fungal genera, LEfSe analysis showed that there was higher relative abundance of *Schizothecium*, *Entoloma*, *Preussia*, *Lecanicillium,* and *Bipolairs* in T1, whereas there was a higher relative abundance of *Thanatephorus*, *Scopulariopsis*, *Fusarium*, and *Conocybe* in CK1 (Fig. 3a) for the flowering stage. In the fruiting stage, *Psathyrella*, *Filobasidium*, *Aphanoascus*, *Cladosporium*, *Microascus*, and *Scopulariopsis* were found in CK2, while only *Schizothecium* was found in T2 (Fig. 3b). Furthermore, the second prevailing genus, *Fusarium,* accounted for 9.70% of all fungal genera in CK1 (only 0.64% in T1). The relative abundance of *Fusarium* was higher in CK2 than in T2 (see Supplementary Table S4).

### Microbial community variety and the link between genera abundance and environmental conditions

**Figure 4:**
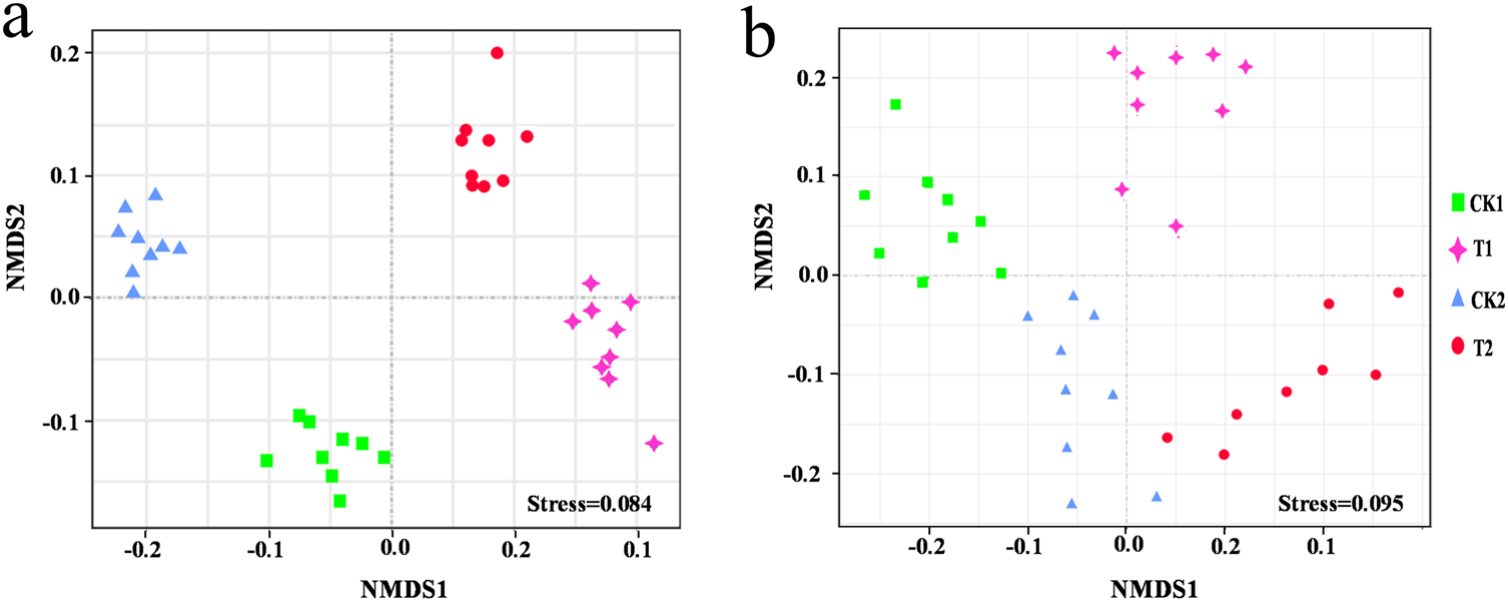
Non-metric multidimensional scaling (NMDS) according to the Euclidean distance plot of bacterial (**a**) and fungal (**b**) microbiota in the flowering stage (CK1 and T1) and fruiting stage (CK2 and T2).

**Figure 5:**
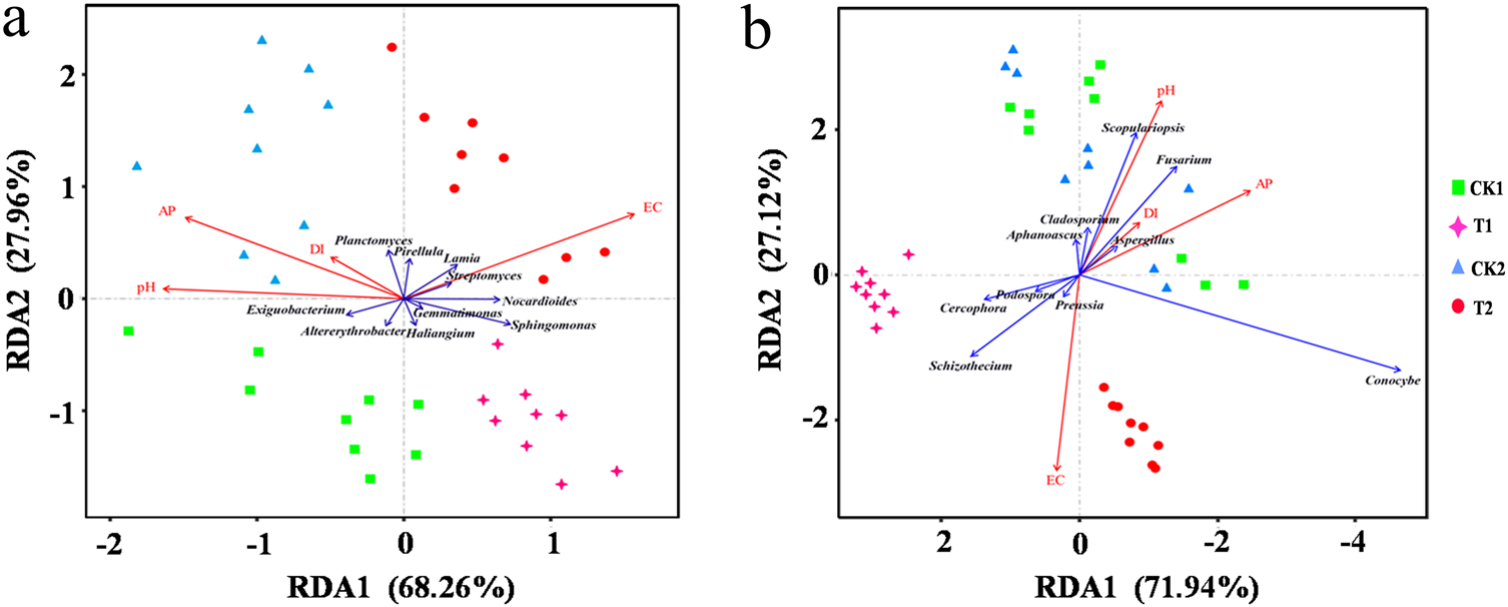
Plots of redundancy analysis (RDA) ordination displaying the interactions between the top 10 bacterial (**a**) and fungal genera (**b**) and soil environmental variables. AP denotes available phosphorus; pH denotes the solar pH; EC denotes electrolyte conductivity; the disease index (DI) denotes healthy plants as “0”and Fusarium wilt plants as “1”. CK1 represents the soil without wheat straw addition at the watermelon flowering stage while T1 represents the soil with wheat straw addition at the watermelon flowering stage; CK2 represents the soil without wheat straw addition at the watermelon fruiting stage and T2 represents the soil with wheat straw addition at the watermelon fruiting stage.

Non-metric multidimensional scaling (NMDS) clearly indicated that there were considerable variations in the composition of the soil bacterial populations between the samples with (T1 and T2) and without (CK1 and CK2) wheat straw addition in the consecutive watermelon monoculture system in the two growth stages evaluated (Fig. 4a). In the NMDS plot, the nine replicates in the same groups were not closely located for the fungal communities in all the samples, which indicated that there was no distinct difference in fungal community composition between two treatments for the two growth stages (Fig. 4b). RDA revealed that the relative abundances of *Planctomyces*, *Pirellula*, and *Exiguobacterium* were positively correlated with the DI for bacteria (Fig. 5a and Supplementary Table S5a). The relative abundances of *Aspergillus*, *Fusarium*, *Sopulariopsis*, *Cladosporium*, and *Aphanoascus* were positively correlated with the DI for fungi (Fig. 5b and Supplementary Table S5b).

### Fungal community network analysis

**Figure 6:**
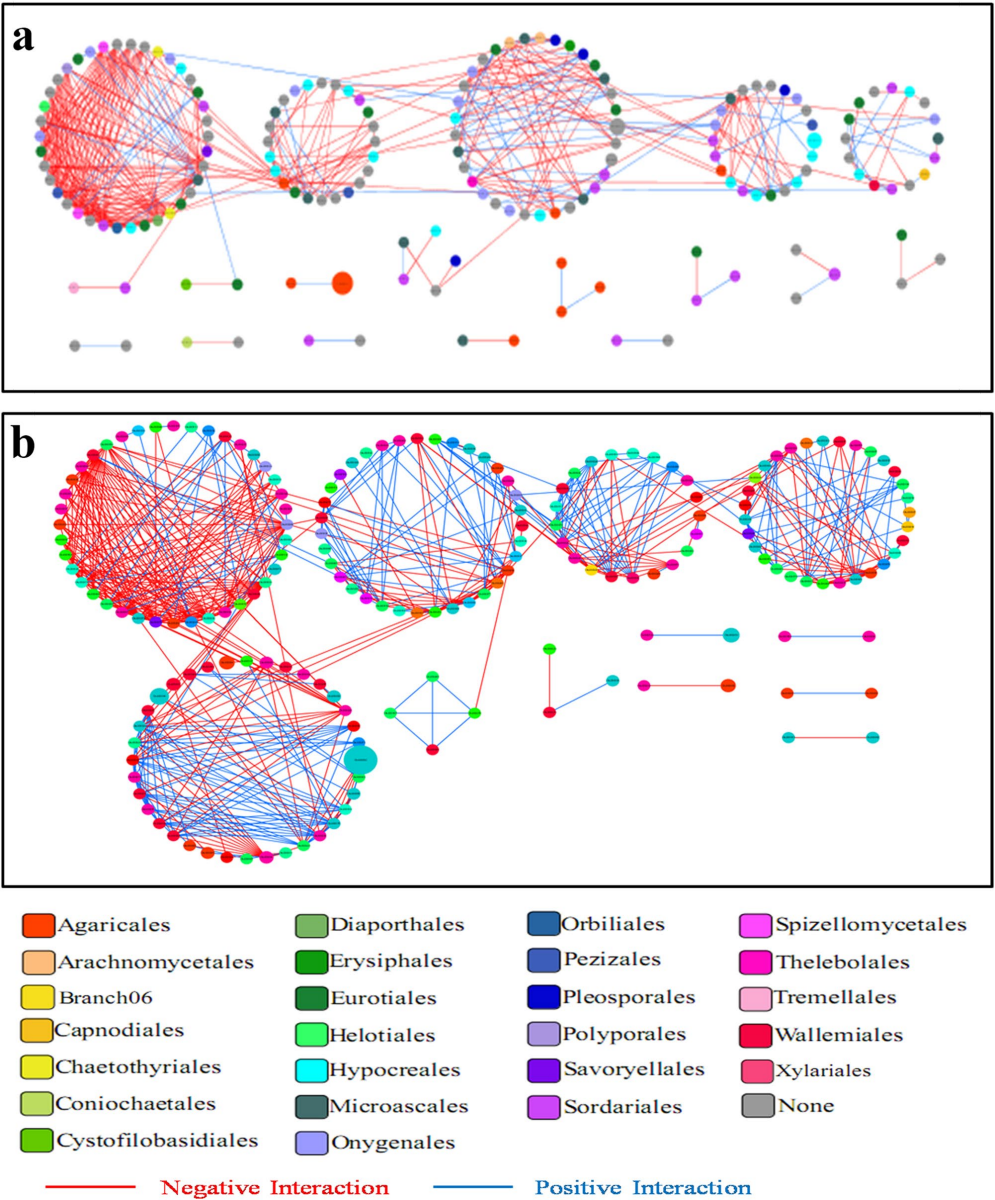
Network plots of fungal community at the order level from soil without (CK1) (**a**) and with (T1) (**b**) wheat straw addition at the watermelon flowering stage. The size of the node corresponds to the relative abundance of the OTUs. The node colors show various phylogenetic associations. Node (edge) connection lines represent co-occurrence with positive (blue) and negative (red) correlations.

Soil microbial network analysis is widely performed to understand the taxonomic and functional relations within complex microbial communities^13^. With respect to fungi, the top 300 OTUs of T1 and CK1 soil at the watermelon flowering stage were chosen for pMEN analysis (Fig. 6). The T1 network consisted of 180 nodes (OTUs), 1,036 connections, and 12 modules, with an average connectivity of 11.511, average path length of 2.999, and clustering coefficient of 0.278. The CK1 network consisted of 166 nodes, 741 connections, and 18 modules, with an average connectivity of 8.927, average path length of 2.920, and clustering coefficient of 0.155. The modularity proportion was higher in the T1 network, although fewer total modules were recognized (Table 1). Strikingly, there were more links in T1 soil (1,036 links) than in CK1 soil (741 links). The positive link/negative link ratio (P/N) was higher in T1 soil (P/N = 0.333) than in CK1 soil (P/N = 0.211), demonstrating that the T1 soil had more complex and positive co-occurrence correlations than the CK1 soil.

**Table 1:** Major topological properties of the empirical phylogenetic Molecular Ecological Networks (pMENs) of fungal communities for soil with (T1 and T2) and without (CK1 and CK2) wheat straw addition and their associated random pMENs.

	Empirical networks									Random networks^d^		
	Soil traits	NO. of original OTUs^a^	Similarity threshold (St)	Network size (n)^b^	R^2^ of Power law	Avg connect (avg K)	Avg path length (GD)^c^	Avg clustering coefficient (avg CC)	Modularity (no. of modules)	Avg path distance (GD)	Avg cluster coefficient (avg CC)	Modularity (M)
Flowering stage	CK1	300	0.89	166	0.719	8.928	2.920	0.155	0.279 (18)	2.700 ± 0.034	0.179 ± 0.011	0.236 ± 0.006
	T1	300	0.86	180	0.645	11.511	2.999	0.278	0.300 (12)	2.536 ± 0.030	0.235 ± 0.014	0.198 ± 0.005
Fruiting stage	CK2	300	0.87	181	0.714	8.166	3.186	0.264	0.409 (15)	2.778 ± 0.029	0.137 ± 0.013	0.266 ± 0.005
	T2	300	0.88	202	0.636	12.545	2.727	0.214	0.261 (14)	2.496 ± 0.026	0.253 ± 0.010	0.186 ± 0.004

^a^The number of OTUs that were originally used for network construction using the random matrix theory (RMT)-based approach.

^b^The number of OTUs (i.e., nodes) in the network.

^c^*GD* geodesic distance.

^d^The random networks were generated by rewiring all of the links of a pMEN with the identical numbers of nodes and links to the corresponding empirical pMEN.

The top 300 OTUs of T2 and CK2 soil at the watermelon fruiting stage were also chosen for pMEN analysis (see Supplementary Fig. S3). The T2 network consisted of 202 OTU nodes, 1,040 connections, and 14 modules, with an average connectivity of 12.545, average path length of 2.727, and clustering coefficient of 0.214. The CK2 network consisted of 181 nodes, 739 links and 15 modules, with an average connectivity of 8.166, average path length of 3.186, and clustering coefficient of 0.264 (Table 1). Strikingly, there were more links in T2 soil (1,040 links) than in CK2 soil (739 links), which indicated that the T2 soil had more complex and stable microbial networks than the CK2 soil. In T2 soil, the P/N (P/N = 0.218) was lower than that in CK2 soil (P/N = 0.451), indicating that the T2 soil had more negative co-occurrence relationships in the microbial community than those in CK2 soil.

**Figure 7:**
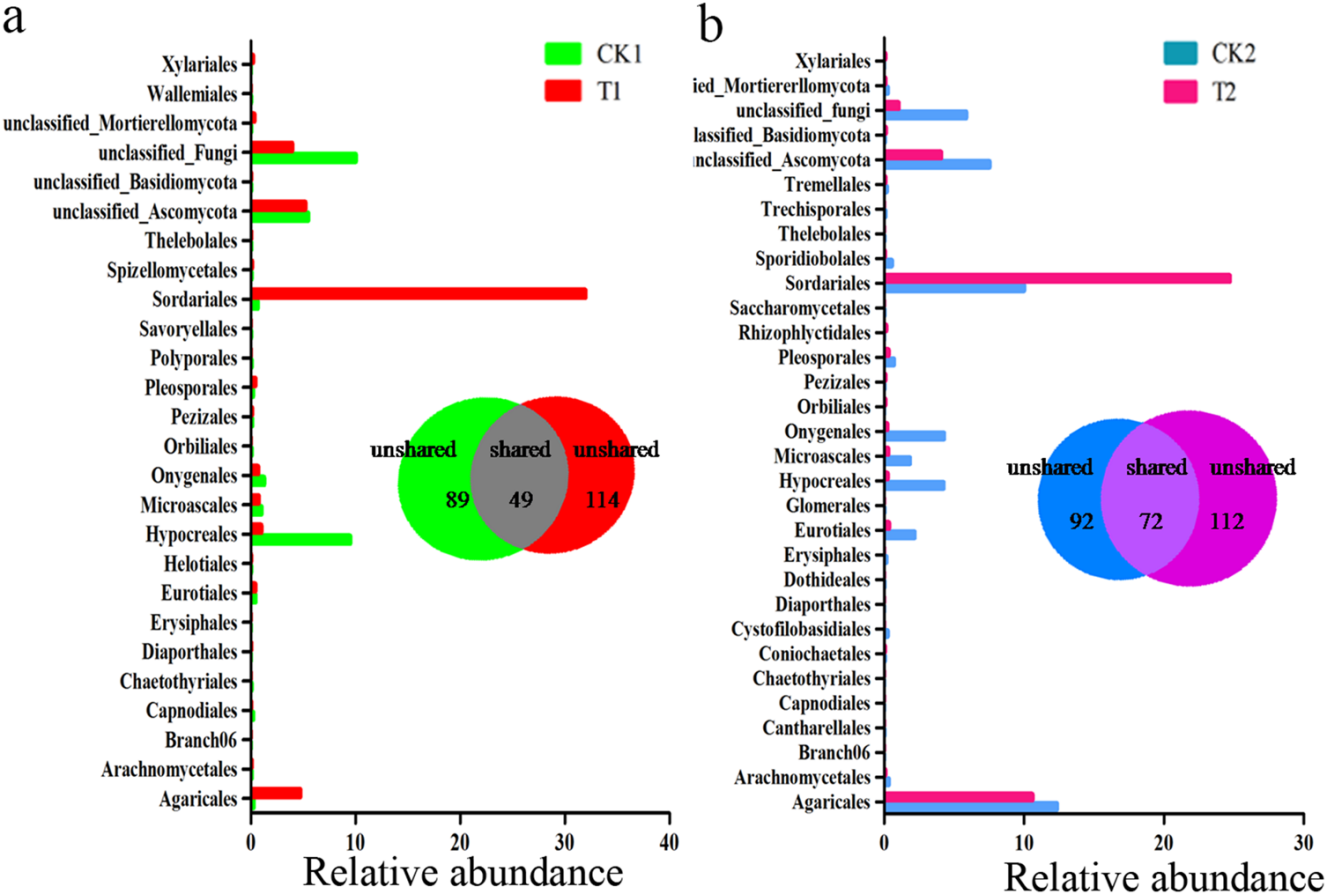
Relative abundance of nodes at the order level in modules inside the fungal network created from the flowering stage (**a**) and fruiting stage (**b**). Venn diagrams display the amount of shared and unshared network nodes in the soil sample with and without wheat straw addition.

In addition, CK1 and T1 networks shared 49 nodes (Fig. 7). Nodes of the *Sordariales*, *Onygenales*, *Microascales*, *Hypocreales*, *Eurotiales*, *Agaricales,* and *Arachnomycetales* genera dominated in both networks. The relative abundance of *Sordariales* and *Hypocreales* was higher in the two networks. Furthermore, there was a higher proportion of *Sordariales-*affiliated OTUs and a lower proportion of *Hypocreales*-affiliated OTUs in T1 compared to CK1 (Fig. 7a). However, 72 nodes were shared between CK2 and T2 networks. Nodes belonging to the *Sordariales*, *Pleosporales*, *Onygenales*, *Microascales*, *Hypocreales*, *Eurotiales,* and *Agaricales* genera dominated in both networks. *Sordariales*, *Hypocreales,* and *Agaricales* were relatively more abundant in these two networks. However, the relative abundance of *Sordariales* and *Hypocreales* had more significant differences in CK2 compared to T2. There was also a higher proportion of *Sordariales*-affiliated OTUs and a lower proportion of *Hypocreales*-affiliated OTUs in T2 compared to CK2 (Fig. 7b). These network analysis results suggested that *Sordariales* dominated in the T1 and T2 soils, which were treated with wheat straw, while *Hypocreales* dominated in the soil (CK1 and CK2) without wheat straw addition.

### Bacterial community network analysis

T1 and CK1 soil bacterial community analysis revealed similar sized networks with 224 and 221 nodes, respectively (see Supplementary Fig. S4 and Table S6). The average connectivity for the T1 and CK1 networks was 7.482 and 7.493, with an average path length of 4.273 and 3.557, respectively. The average clustering coefficient value (0.323 or 0.326) was comparable in the T1 and CK1 soil networks, while modularity was somewhat lower in the T1 network (0.403) than in the CK1 network (0.440). However, the number of modules in T1 (27) was higher than that in CK1 (22). In T1 and CK1 soil, the total number of links was 828 and 838 (P/N = 2.33 for T1 soil and P/N = 2.37 for CK1 soil), respectively. However, T2 and CK2 soil had different nodes (228 and 201, respectively) at the watermelon fruiting stage. Furthermore, the average connectivity was higher in T2 (4.263) than in CK2 (3.562) networks. The average path length, average clustering coefficient value, and modularity were higher in CK2 than in T2 networks (see Supplementary Fig. S5 and Table S6). In T2 and CK2 soils, the total number of links was 486 and 358 (P/N = 1.612 for T2 soil and P/N = 1.732 for CK2 soil), respectively. The data suggested that wheat straw addition did not affect the bacterial co-occurrence relationship in both the watermelon flowering and fruiting stages.

Inside the T1 versus the CK1 network, a greater percentage of OTUs associated with *Proteobacteria*, and a reduced OTU ratio for *Chloroflexi, Bacteroidetes,* and *Acidobacteria* were found. (see Supplementary Fig. S6a)**.** However, a higher proportion of *Proteobacteria* and *Actinobacteria*- affiliated OTUs and a lower proportion of *Gemmatimonadetes*, *Euryarchaeota*, *Chloroflexi*, and *Bacteroidetes*-affiliated OTUs inside the modules were identified in the T2 versus CK2 network (see Supplementary Fig. S6b).

## Discussion

Fusarium wilt is a soil-borne fungal illness that has a great influence on economic crops, such as cucumber^25^^,^ watermelon^26^^,^ melon^27^^,^ banana^28^^,^ and vanilla^14^. Monoculture cropping is one of the most important factors that lead to the development of soil-borne diseases. It has been widely reported that crop straw can benefit the management of soil-borne diseases and reduce the use of fertilizers and pesticides. In previous studies, root and stem rot in cucumber plants were found to be controllable with the incorporation of lettuce to the system, whereas grape residue and garlic straw were demonstrated to inhibit Fusarium wilt and root-knot nematodes in tomato plants, respectively^29–31^. In the present study, the results showed that in the consecutive watermelon monoculture system, adding wheat straw did not substantially affect soil bacterial diversity but changed the fungal diversity at the two stages evaluated (see Supplementary Table S1). The DI was closely related to the existence of *Planctomyces*, *Pirellula*, and *Exiguobacterium* in the bacterial community and *Aspergillus*, *Fusarium*, and *Sopulariopsis* in the fungal community (Fig. 5). But we don't know that they are pathogens or beneficial. The verification is required in further.

Plant species, soil types, and root exudates can affect soil microbial OTUs in the entire community^26,32,33^. Soil microbial taxonomic composition apparently changed after wheat straw addition in the two growth stages (Fig. 1). *Proteobacteria*, *Actinobacteria*, and *Gemmatimonadetes* were the most abundant phyla in all the samples (Fig. 1a). However, *Actinobacteria*, *Chloroflexi*, and *Saccharibacteria* were more abundant in the soil with added wheat straw (Fig. 1a). Previous studies have also found that *Rhizoctonia*-suppressive soil had a higher abundance of *Actinobacteria*^34^. *Actinobacteria* is known to secrete a large number of secondary metabolites to inhibit the growth of pathogens while promoting plant growth^35^. For example, *S. lividans* can suppress *Verticillium dahliae* growth by producing prodiginies^36^. *S. alboflavus* TD-1 can suppress the growth of storage disease fungal pathogens, such as *Fusarium moniliforme*, *Aspergillus flavus*, and *Penicillum citrinum* through the production of volatile compounds^37^. Zhao et al.^38^ found that *S. bikiniensis* HD-087 could activate systemic resistance to *F. oxysporum* f. sp. *cucumerinum* in cucumber. We speculated that *Chloroflexi* and *Saccharibacteria* may also play important roles in disease suppression. For the fungal community, *Ascomycota* was more abundant in the samples with wheat straw addition than in the samples without wheat straw during the flowering stage. The relative abundance of *Ascomycota* with wheat straw later decreased in the fruiting stage. *Ascomycota* is a large and diverse group of fungi that play an important role in maintaining the function of the soil ecosystem. For example, *Preussia*, *Penicillium* and *Chaetomium* belong to the *Ascomycota* family. *Preussia* can inhibit fungal diseases, while *Penicillium* and *Chaetomium* can degrade organic matter^39–41^. The result was also in line with an earlier report that *Ascomycota* is the primary phylum in wheat monoculture^42^. Our findings suggested that these phyla could play comparable roles in inhibiting Fusarium wilt in watermelon.

LEfSe assessment showed that watermelon Fusarium wilt disease-suppression was associated with some specific microbial genera. For bacteria, the *Actinobacteria* phylum reads were very different between the soil samples with and without wheat straw. *Sporichthya*, *Aeromicrobium*, *Nonomoricola*, *Marmoricola*, *Ilumatobacter*, *Streptomyces*, *Agromyces*, *Patulibacter*, *Amycolatopsis*, and *Nocardioides* were part of the *Actinobacteria* phylum in T1 and comprised 4.30% of the total bacterial readings. Additionally, the *Actinobacteria* phylum showed higher abundance in the T2 soil than in the CK2 soil (Fig. 2).

For fungi, the most abundant genus in CK1 soil was *Fusarium* (see Fig. 3 and Supplementary Table S4). Earlier studies showed that some *Fusarium* species were the pathogens and important indicators of Fusarium wilt disease in the watermelon monoculture system. Furthermore, abundance analysis of FON1 at the two growth stages showed that the FON1 population was higher in CK1 than in T1 and higher in CK2 than in T2 (see Supplementary Fig. S7). Altogether, our results confirmed that wheat straw affected the soil-associated microbial community structure and abundance in the consecutive monoculture watermelon system, leading to the abundance increase of beneficial bacterial microorganisms and the abundance decrease of harmful fungal microorganisms such as *Fusarium*. Hence, it is possible that the reduction of the watermelon Fusarium wilt disease was due to the increase of beneficial microorganisms in the soil after wheat straw addition.

Microbial network assessment was conducted to compare the diversity of soil microbial communities with and without wheat straw addition in the continuous monoculture cropping system. The microbial molecular ecological network analysis was performed using the top 300 abundant genera in the soil microbial communities for two growth stages. For the bacterial network, the total nodes were not considerably distinct in connectivity and clustering between the two soil networks with and without wheat straw addition at the two growth stages. The shared nodes also did not differ considerably between soil with and without wheat straw at the two growth stages (see Supplementary Table S6).

For the fungal network, there were more nodes in the soil with wheat straw addition at the two growth stages (Table 1). The members were more interrelated in the network, in which the community might function better for the soil and plant health, although the specific functions are unknown. Interestingly, *Sordariales* held a dominant position in both T1 and T2 while *Hypocreales* held a dominant position in CK1 and CK2 (Fig. 7). The results were consistent with that for the fungal network in the healthy and disease soil in the potato monoculture system^43^. Future studies will be needed to identify the particular roles of these microbial species in improving the control of Fusarium wilt disease, either through direct pathogenic inhibition or host resistance activation. The soil with added wheat straw showed an increased level of positive co-occurrence associations for the fungal network compared to the soil without wheat straw at the two growth stages evaluated. Zhang et al.^44^ showed that more positive microbial community interactions may induce more collaborations related to increased community functions, although these interactions can also reduce the stability of the community^45^. Furthermore, soil with added wheat straw had higher average connectivity than that without wheat straw for fungal networks (Table 1).

The average connectivity of soil with and without wheat straw addition was similar for the bacterial networks (see Supplementary Table S5). Scheffer et al.^46^ reported that a tightly connected network may be more resistant to disturbance. Moreover, soil microbial interactions may contribute directly to a decrease in plant diseases by creating coexistence, according to Bever et al.^47^. In this study, we showed that there were significant complex connections among fungal communities in the soil with and without wheat straw addition, but not among the bacterial communities.

NMDS and RDA analyses demonstrated that soil microbiota had significantly different compositions in the soil with and without wheat straw addition at two growth stages (Figs. 4, 5). The relationships of the top ten fungal and bacterial genera and soil environmental variables are presented in RDA order plots (Fig. 5). Previous studies also showed that the most prosperous growth for *Fusarium oxysporum* occurred at pH values between 6 and 7^48^. The normal EC value of crop growth is 0.4–1 mS/cm and too low or too high EC is not conducive for plant growth. We found that soil AP, EC, and pH were associated with soil microbiota composition and also had close relationships with DI (Fig. 5). Some previous reports showed that *Sphingomonas* could promote crop resistance against multiple pathogens^49^. The *Gemmatimonas* genus has been shown to be related to the formation of soil organic content and has a significant function in promoting the degradation of cellulose^50^.

Though the *Fusarium* genus includes some pathogens, the enhanced abundance of other co-generic species can also be correlated with the elevated incidence of diseases^51^. In order to determine the particular roles of these species in developing the Fusarium wilt disease, further research will be needed. In our study, *Fusarium*, *Scopulariopsis*, *Cladosporium*, *Aphanoascus*, *Aspergillus,* and *Conocybe* were positively related to DI, whereas *Preussia*, *Podospora*, *Cercophora,* and *Schizothecium* were negatively correlated with DI (Fig. 5). In addition, we found that *Schizothecium* and *Preussia* were the dominant genera in soil with wheat straw according to LEfSe analysis (Fig. 3). Mapperson et al*.*^52^ reported that *Preussia* sp. have antimicrobial activity. Thus, we speculated that *Schizothecium* might play a key role in inhibiting the watermelon Fusarium wilt. In addition, *Schizothecium* might degrade the wheat straw and the product could also inhibit the watermelon Fusarium wilt. Our team is currently studying the effects of wheat straw degradation products on pathogens. Several studies have demonstrated that wheat straw addition is an efficient soil-borne disease prevention strategy^53^. However, it is not clear whether the microorganisms can directly inhibit the pathogen or whether wheat straw degradation inhibits the pathogen to reduce watermelon Fusarium wilt. For example, Chu et al.^54^ found that exogenous palmitic acid could control FON1 and promote watermelon growth. Syringic acid changed cucumber rhizosphere microorganisms and exerted negative effects on cucumber seedling growth^55^. Therefore, we will devote future studies to the effect of chemicals that mediate diseases. In this study, we confirmed that wheat straw addition alone is an efficient, eco-friendly, safe, and durable technique for managing watermelon wilt disease caused by Fusarium wilt in the monoculture cropping system.

## Conclusion

In conclusion, our results showed that wheat straw addition could increase the relative abundance of *Chloroflexi*, *Saccharibacteria,* and *Schizothecium*, but reduce the relative abundance of the pathogen *Fusarium*. We speculated that *Schizothecium* may play particularly important roles in reducing the incidence of the watermelon Fusarium wilt. In addition, wheat straw significantly affected the fungal network structure but had little effect on the bacterial network structure. We also found that there was higher abundance of *Sordariales* and lower abundance of *Hypocreales* in soil communities with wheat straw in the consecutive watermelon monoculture system. We concluded that wheat straw addition to the soil could enhance the resistance of watermelon plants against Fusarium wilt pathogens by affecting the soil fungal communities and functions in the consecutive watermelon monoculture system. Our study demonstrates that the application of wheat straw is an efficient and sustainable approach to control destructive soil-borne pathogens and diseases, improve the plant yield and health, and protect the environment by reducing chemical applications.

## Methods

### Experimental materials and conditions

Three wheat straws (X00, X01, and X03), one rice straw, and one maize straw, were supplied by the Vegetable Physiological Ecology Laboratory at the College of Horticulture and Landscape Architecture at Northeast Agricultural University. The root exudates of X00 could inhibit the growth of FON, but had no significant effect on the growth of watermelon. The root exudates of X01 could inhibit FON growth and significantly promoted watermelon growth. The inhibitory capacity of X03 root exudates on FON was weaker than that of X00 and X01 and had no effect on the growth of watermelon.

The five straws were naturally air dried and crushed to pieces with approximate lengths of 0.5–1 cm. Seeds of the watermelon [*Citrullus lanatus* (Thunb.) Matsum. and Nakai var. Lanatus] cultivar Zaojia 84–24, which is susceptible to Fusarium wilt, were provided by the Xinjiang Academy of Agricultural Sciences. At the Xiangfang Farm of Northeastern Agriculture University (about 44° 04′ N and 125° 42′ E in Harbin, China), monocultivated soil was collected from the surface layer (0–20 cm) of watermelon cultivar continuously grown for 5 years and affected by *Fusarium oxysporum* f. sp. *niveum* race 1 (FON1). The monocultivated soil was mixed well and sent to the lab. This monocultural soil, with a pH of 7.11 and an electrolytic conductivity (EC) of 0.28 mS/cm, contained 36.40 g/kg of organic matter. The inorganic substances consisted mainly of alkaline hydrolytic nitrogen at 139.00 mg kg, total phosphorus at 3.10 g/kg with 271.80 mg/kg of available phosphorus (AP), and total potassium at 46.40 g/kg with 221.50 mg kg of available potassium (AK).

### Experiment design

The pot experiments were conducted from April to July 2018 in a greenhouse located in the Experimental Center of Northeast Agricultural University, Harbin, China. The day/night average temperature was about 25 °C/18 °C and the average relative humidity was about 60% in the greenhouse from April to May. From June to July, the day/night average temperature was about 32 °C/25 °C and the average relative humidity was about 70–80% in the greenhouse. Watermelon seeds were soaked for 30 min at 55 °C, followed by three to four rinses with sterile distilled water and germination in a peat: perlite medium (1:1 v/v) after growing in the Experimental Center greenhouse of Northeast Agricultural University.

The experiments consisted of six treatment groups in which watermelon seedlings with four true leaves were transplanted into a plastic pot (18 cm in diameter and 18 cm in height) filled with 5 kg monoculture watermelon soil containing 0 g of crop straw (CK), 50 g of X00 wheat straw (X00), 50 g of X01 wheat straw (X01), 50 g of X03 wheat straw (X03), 50 g of rice straw (rice), or 50 g of maize straw (maize). Each treatment was administered to 40 pots and was repeated three times. The arrangement of the pots was completely randomized and the plants were subjected to typical management procedures and equal amounts of irrigation. The disease incidence and severity were observed and calculated at the flowering stage (approximately 30 days after transplanting).

### Measurement of the incidence and severity of watermelon Fusarium wilt

To evaluate the effects of five straws (rice, maize, X00, X01, and X03) on the incidence and severity of watermelon Fusarium wilt during the flowering period, pot culture experiments were carried out in a greenhouse. The wilt incidence was expressed as the proportion of symptomatic plants in the total pot cultures. The disease severity was scored according to the proportion of wilting regions in the leaves or stems in the entire plant following a visual estimation: 0, the whole plant was healthy; 1, about 25%; 2, about 50%; 3, about 75%; 4, the whole plant was wilted and dead. The disease index (DI) was designated for healthy plants as “0” and for Fusarium wilt-infected plants as “1”.

### Soil sample collection

Based on the results shown in Supplementary Fig. S1, the wheat X01 was chosen as the material to evaluate the soil microbial community and network changes. The experiments involved two treatments (T1 and T2) and two controls (CK1 and CK2). T1 and CK1 were the experiments at the watermelon flowering stage (approximately 30 days after transplanting) and T2 and CK2 were experiments at the watermelon fruiting stage (approximately 60 days after transplanting). The rhizosphere and bulk soil associated with the watermelon plants were collected at the flowering and fruiting stages in the spring of 2018. Based on the protocol established by Song et al*.*^56^ soil samples were collected from five plants for each repetition. In order to reduce the errors and visualize the microbial network, nine replicates were performed. Briefly, the roots of watermelon seedlings were carefully harvested and loosely attached or tightly adhered soil was shaken off or mildly separated. The rhizosphere soils were passed through a 2-mm mesh sieve and were stored at − 70 °C for microbial DNA extraction in sterile plastic bags filled with ice. For the soil samples, five watermelon rhizosphere soil samples were pooled as a single sample with a total of nine repetitions. For physicochemical analysis, the bulk soil was collected and sieved through a 2-mm sieve followed by thorough homogenization and air-drying.

### Analysis of physical and chemical soil properties

The soil pH and electrolyte conductivity (EC) were measured with a glass electrode and a conductivity meter in soil–water suspension (1:2.5 w/v) according to the method developed by Bao^57^. The available phosphorus (AP) in the soil was extracted with 0.5 M NH4^+^OAc (pH = 7) and a Continuous Flow Analyzer (SAN^++^, Skalar, Breda, Netherlands) was used to analyze the soil filtrates.

### DNA extraction and amplicon sequencing

The microbial DNA of soil samples was extracted with the E.Z.N.A. stool DNA Kit (Omega Biotek, Norcross, GA, U.S.). The bacterial 16S rDNA V3-V4 hypervariable regions were PCR-amplified with the primers 341F: CCTACGGGNGGCWGCAG and 806R: GGACTACHVGGGTATCTAAT. The eukaryotic internal transcribed spacer (ITS) regions of the fungi were PCR-amplified with the primers ITS3_KYO2F: GATGAAGAACGYAGYRAA and ITS4R: TCCTCCGC TTATTGATATGC.

The expected PCR amplifications were performed in triplicate in 50 μL reaction mixtures composed of 5 μL 10 × Buffer, 5 μL dNTPs of 2.5 mM, 1.5 μL of each primer at 5 μM, 1 μL polymerase, and 100 ng template DNA. The target fragments were amplified using the following parameters: initial denaturation at 95 °C for 2 min, 35 cycles of 98 °C for 15 s, 63 °C for 30 s, and 68 °C for 30 s, followed by a final extension at 68 °C for 10 min. The amplicon products were loaded onto 2% pre-stained agarose gel (Thermo Scientific) and the expected fragments were extracted and quantified using AxyPrep DNA Gel Extraction Kit (Axygen Biosciences, Union City, CA, US) and QuantiFluor-ST (Promega, US) following the manufacturer’s protocol. The DNA was end-repaired, dA-tailed, ligated to Illumina paired-end adaptors and amplified using PCR with 500 bp inserts for library construction. The quality and concentration of the final libraries were verified and determined with the Agilent 2,100 Bioanalyzer (Agilent Technologies Co. Ltd, USA) and KAPA Library Quantification Kits (Kapa Biosystems, USA), respectively. Amplicons were purified, normalized, and pooled for 2 × 250 sequencing on the Illumina MiSeq platform (Illumina, San Diego, USA) (see Supplementary Table S7).

### Measurement of FON1 population in rhizosphere soil

The FON1 populations in the soil samples were quantified by real-time PCR (Analytik Jena AG, Germany) and the primers fon-1 (5′-CGATTAGCGAAGACATTCACAAGACT-3′) and fon-2 (5′-ACGGTCAAGAAGATGCAGGGTA AAGGT-3′) were used to identify FON1^58^. Real-time PCR was performed in a 20 µL reaction system containing 10 µL of 2× Real SYBR Mixture (TIANGEN Biotech, China), 0.5 µL of each primer, and 2 µL of DNA. The PCR was processed at 94 °C for 5 min, 35 cycles of 95 °C for 30 s, 54 °C for 30 s, and 72 °C for 30 s, followed by extended elongation at 72 °C for 3 min with a Real Time PCR system (Analytik Jena AG, Germany). The quantification references were generated from a cloning plasmid carrying the FON1 gene and the standard curve was generated as described by Whelan et al.^59^. The signal threshold was automatically set by the system and all real-time PCR reactions were carried out in technical triplicates.

### Bioinformatics pipelines

The raw sequencing reads were filtered using FASTQ^60^ and the paired end reads with a minimum of 10 bp overlap and a maximum of 2% mismatch error rates were merged using FLSAH^61^ (v1.2.11) as raw tags. QIIME^62^ (V1.9.1) was further applied to remove chaotic sequences^63^. The resulting clean reads were aligned with the reference database (https://drive5.com/uchime/uchime_download.html) using the UCHIME algorithm (https://www.drive5.com/usearch/manual/uchime_algo.html). UPARSE grouped the tags with 97% identity into operational taxonomic units (OTUs). The sequences with maximum abundance were chosen in each cluster as the reprehensive sequences. A Venn diagram was constructed using the VennDiagram version 1.6.20 package in R to define the distinctive and common OTUs between groups. By using RDP classifier (Version 2.2), the representative sequences were grouped into organisms by a naive Bayesian model based on the SILVA Database (https://www.arb-silva.de/)^64^.

The fungal and bacterial alpha diversity calculated in QIIME were estimated with the Chao1, ACE, Shannon, and Simpson indexes^62^. The Alpha index comparison between groups was quantified with a Welch's t-test and a Wilcoxon rank test in R. The Alpha index comparison among groups was analyzed with a Tukey’s HSD test and a Kruskal–Wallis H test in R (https://www.R-project.org). The OTU rarefaction curve was also plotted in QIIME. In order to reveal the microbiome composition and the relationship between environmental factors and microbial abundance, non-metric multidimensional scaling (NMDS) and redundancy analysis (RDA) were performed. NMDS was based on weighted UniFrac metric matrices and was performed to explore the differences in microbial community composition. RDA was performed to examine the relationship among the frequencies of the OTU, samples, and measured soil variables. The linear discriminant analysis (LDA) effect size (LEfSe) method was applied to assess the soil microbiome changes related to the groups with (T1 and T2) and without (CK1 and CK2) wheat straw addition at the flowering and fruiting stages^65^. LEfSe was first performed using the Kruskal–Wallis rank sum test between all the samples, followed by the Wilcoxon rank sum test.

### Network analyses

The interaction network approach with the pMEN was used to analyze the top 300 most enriched bacterium and fungi OTUs in the soil samples^66^ (see Supplement Table S8). Based on the Random Matrix Theory (RMT), the optimal similarity threshold (St) was automatically determined prior to building the network. The Molecular Ecological Network Analysis Pipeline (https://ieg2.ou.edu/MENA/main.cgi) was used for evaluation and the Cytoscape 3.3.0 software was used for network graph visualization.

### Statistical analysis

One-way ANOVA was used to analyze the disease incidence and severity of watermelon Fusarium wilt disease with SAS 9.3 (SAS Institute Inc., Carry, NC, USA). The Student's t-test was used to analyze the soil physiochemical properties, bacterial and fungal taxa (phylum and genus), and microbial alpha diversity indices in the soil. The Venn diagram was obtained with the Venny tool (https://bioinfogp.cnb.csic.es/tools/venny/). Differences at a P value < 0.05 were considered statistically significant.

## Supplementary information

Supplementary Information 1.

Supplementary Information 2.

Supplementary Information 3.

Supplementary Information 4.

Supplementary Information 5.

Supplementary Information 6.

## Data Availability

Raw readings were submitted to the Sequence Read Archive (SRA) for the NCBI database (Accession Number: PRJNA528998).
