# Supplementary material for: Control of Fusarium wilt by wheat straw is associated with microbial network changes in watermelon rhizosphere
Source: Sci Rep. 2020 Jul 29;10:12736. doi: 10.1038/s41598-020-69623-6 (PMC7391731; doi:10.1038/s41598-020-69623-6)
Supplement: Supplementary file 1 — Supplementary Information 1. [file 41598_2020_69623_MOESM1_ESM.docx]

**Control of Fusarium wilt by wheat straw is associated with microbial network changes in watermelon rhizosphere**

Lili Tang^1,2,3^, Ye Xia^5^, Chao Fan^1,2,4^, Jinming Kou^1,2^, Fengzhi Wu^1,2^, Wenhui Li^1,2🖂^, Kai Pan^1,2🖂^


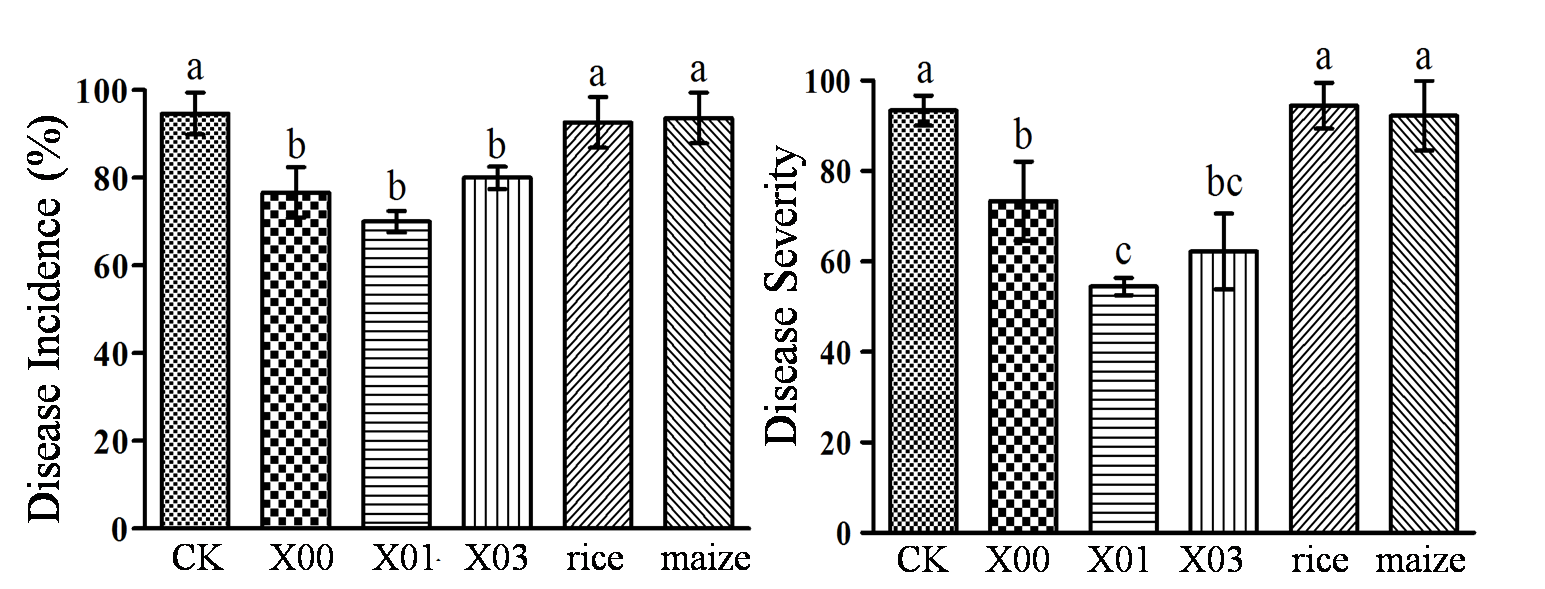


**Fig. S1** Disease incidence (A) and severity (B) of watermelon *Fusarium* wilt. Data represent the means of three replicates and the standard deviation, indicated by vertical bars. According to Tukey's tests, the various tiny letters above the bars represent distinct groups at *P* = 0.05.


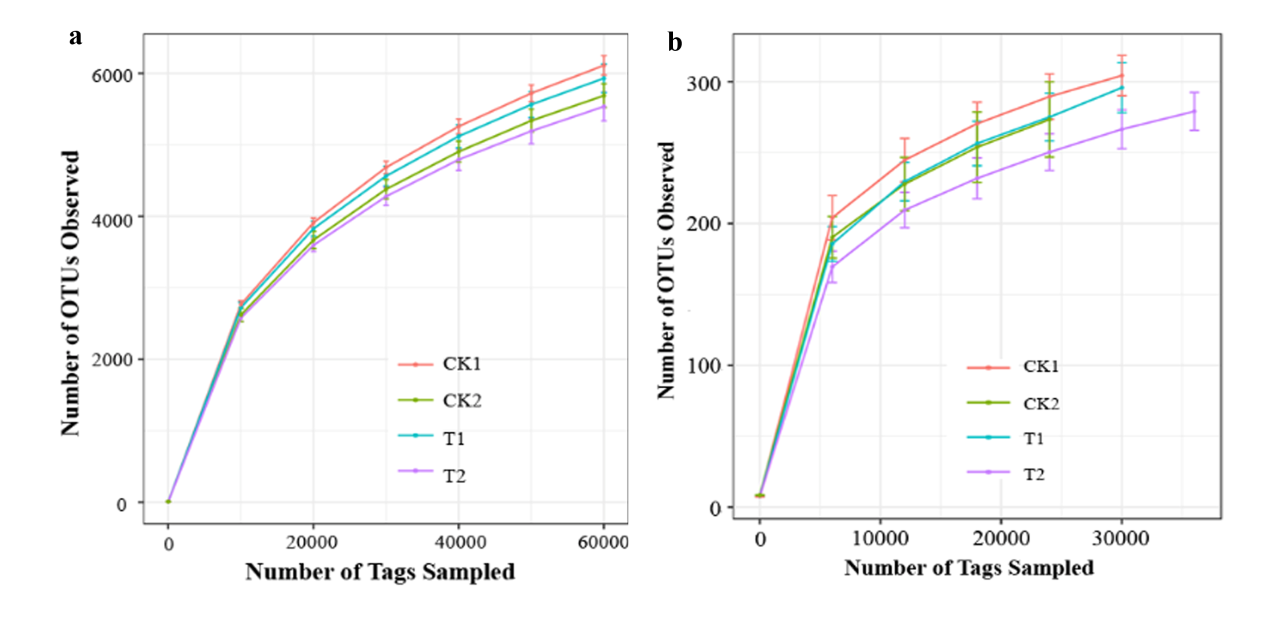


**Fig. S2** Curves of rarefaction for bacterial (A) and fungal (B) communities in the quantity of operational taxonomic units (OTUs). The 97% sequence similarity of OTUs was delineated. CK1 represents the soil without wheat straw addition at the watermelon flowering stage while T1 represents the soil with wheat straw addition at the watermelon flowering stage; CK2 represents the soil without wheat straw addition at the watermelon fruiting stage and T2 represents the soil with wheat straw addition at the watermelon fruiting stage. Bars indicate standard deviation.


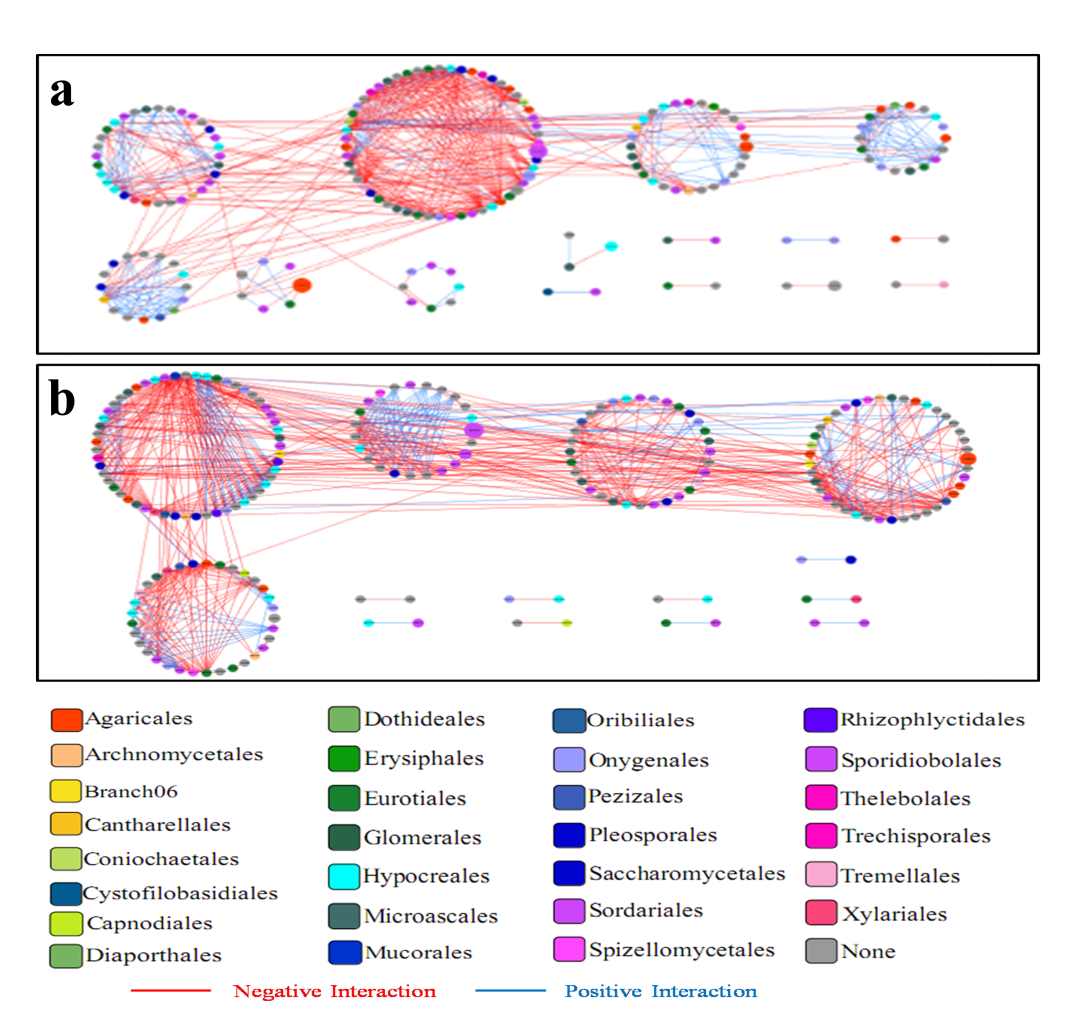


**Fig. S3** Network plots of fungal community from the soil sample without wheat straw addition (CK2) (A) and with wheat straw addition (T2) (B) at the watermelon flowering stage. The size of the node is proportional to the relative abundance of an OTU. The node colors show various phylogenetic associations. Node (edge) connection lines represent co-occurrence with positive (blue) and negative (red) correlations.


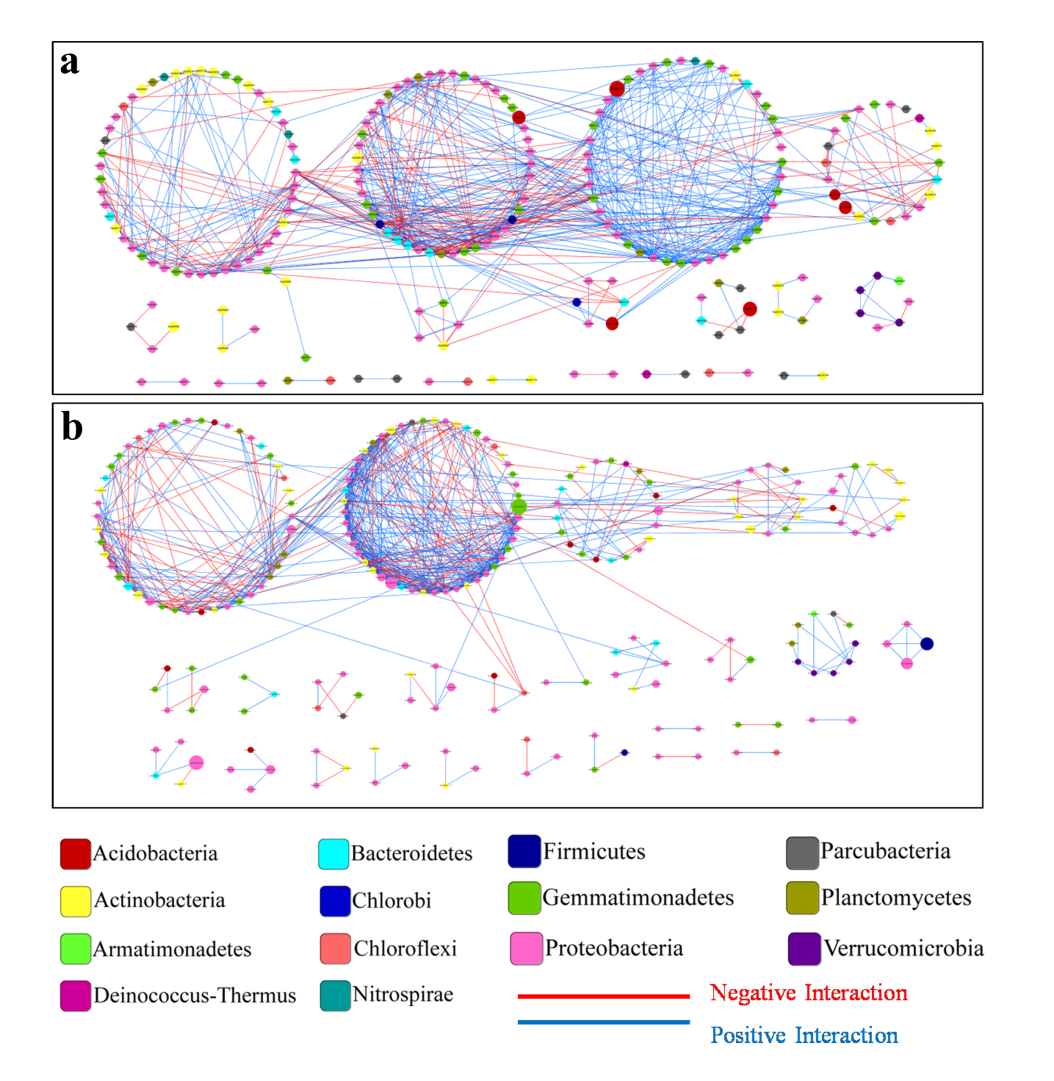


**Fig. S4** Network plots of bacterial community from the soil samples without (CK1) (A) and with wheat straw addition (T1) (B) at the watermelon flowering stage. The size of the node is proportional to the relative abundance of an OTU. The node colors show various phylogenetic associations. Node (edge) connection lines represent co-occurrence with positive (blue) and negative (red) correlations.


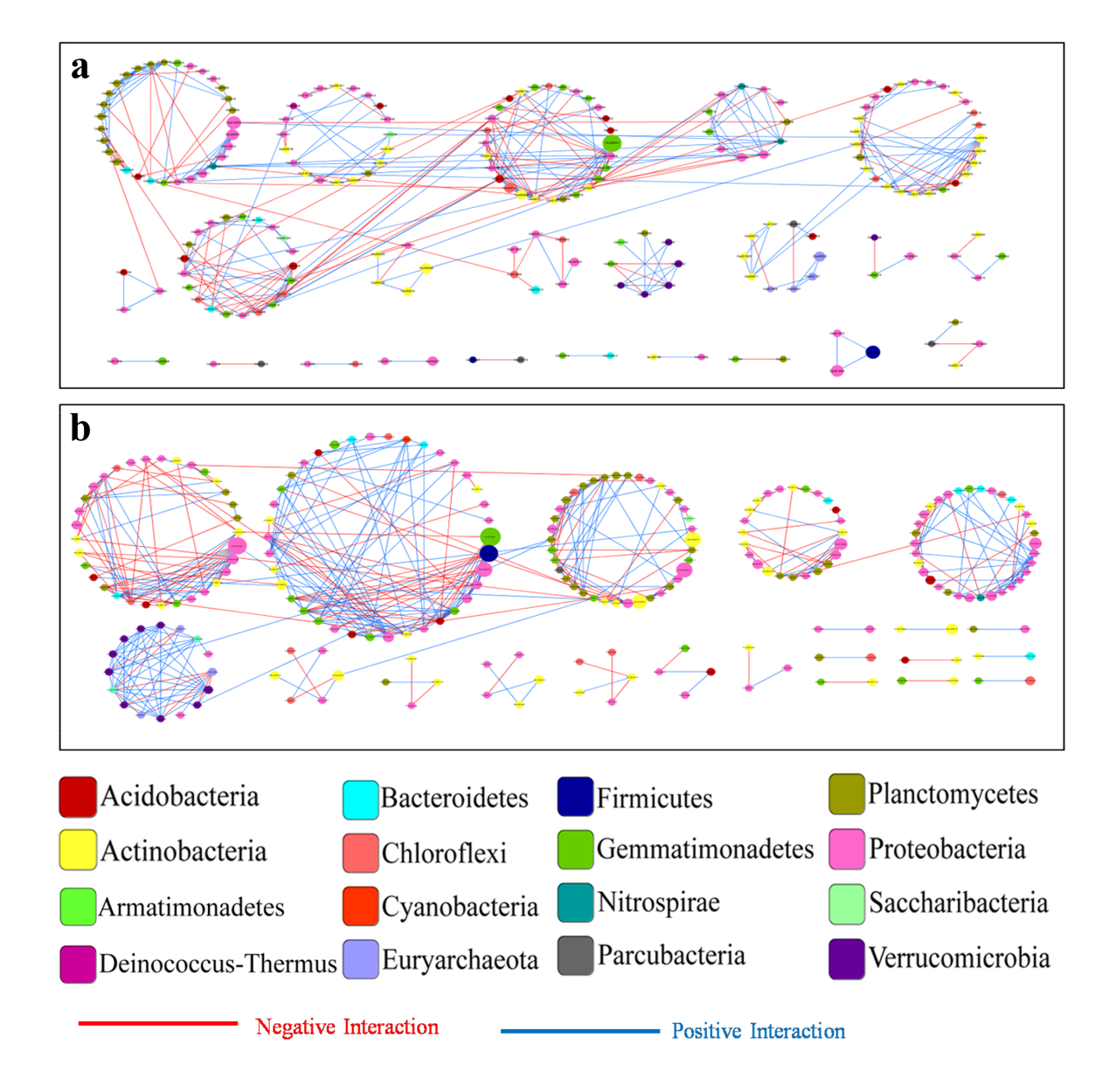


**Fig. S5** Network plots of bacterial community from the soil sample without (CK2) (A) and with (T2) (B) wheat straw addition at the watermelon fruiting stage. The size of the node is proportional to the relative abundance of an OTU. The node colors show various phylogenetic associations. Node (edge) connection lines represent co-occurrence with positive (blue) and negative (red) correlations.

**
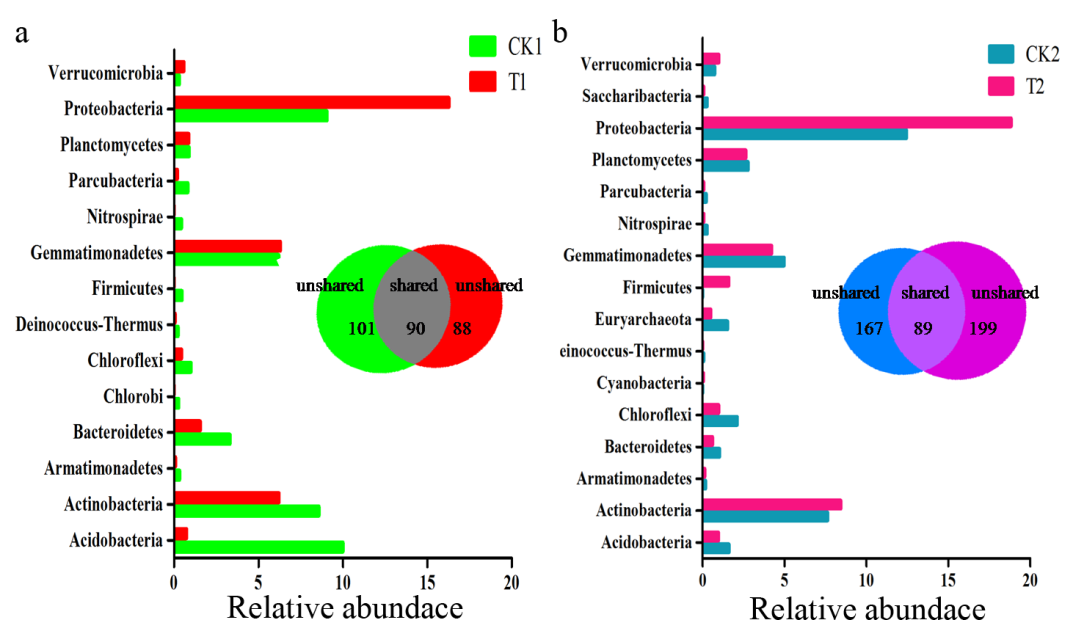
**

**Fig. S6** Relative abundance of nodes at the phylum level in modules inside bacterial network created from the flowering stage (A) and fruiting stage (B). Venn diagrams display the amount of shared and unshared network nodes for the soil samples with and without wheat straw addition.


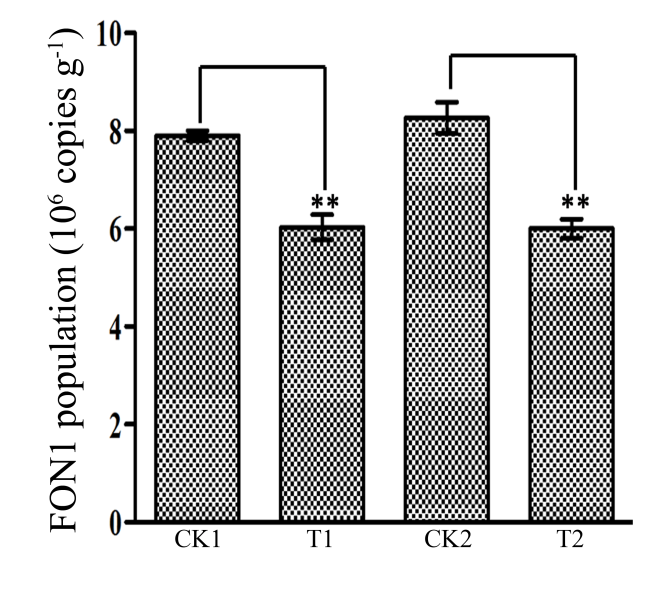


**Fig. S7** The *Fusarium oxysporum* f.sp. *niveum* abundance in the watermelon rhizosphere with and without wheat straw addition in the watermelon monoculture system. ** represents the significance (P < 0.01) between the soil samples with (T1 and T2) and without (CK1 and CK2) wheat straw addition according to the Student's t-test (n = 9). CK1 represents the soil without wheat straw addition at the watermelon flowering stage while T1 represents the soil with wheat straw addition at the watermelon flowering stage; CK2 represents the soil without wheat straw addition at the watermelon fruiting stage and T2 represents the soil with wheat straw addition at the watermelon fruiting stage.

**Table S1a** Diversity and richness indexes of bacterial communities at watermelon flowering stage (CK1 and T1) and fruiting stage (CK2 and T2)

|  | NO. of OTUs | Chao1 | ACE | Shannon | Simpson |
| --- | --- | --- | --- | --- | --- |
| CK1 | 6002±356a | 8957±454a | 8939±470a | 10.43±0.08a | 0.9967±0.0008a |
| T1 | 5785±306a | 8647±408a | 8605±459a | 10.43±0.10a | 0.9971±0.0002a |
| CK2 | 6033±322a | 8203±392a | 8169±391a | 10.30±0.16a | 0.9966±0.0010a |
| T2 | 5630±370a | 7995±517a | 7935±537a | 10.31±0.07a | 0.9970±0.0002a |

**Table S1b** Diversity and richness indexes of fungal communities at watermelon flowering stage (CK1 and T1) and fruiting stage (CK2 and T2)

|  | NO. of OTUs | Chao1 | ACE | Shannon | Simpson |
| --- | --- | --- | --- | --- | --- |
| CK1 | 348±23a | 429±18b | 429±18b | 5.14±0.38a | 0.9254±0.0278a |
| T1 | 367±20a | 459±33a | 461±33a | 5.18±0.17a | 0.9308±0.0143a |
| CK2 | 352±11a | 447±30a | 453±35a | 4.92±0.10b | 0.9602±0.0047a |
| T2 | 374±24a | 451±26a | 455±26a | 5.62±0.15a | 0.9560±0.0064a |

CK1 represents the soil without wheat straw addition at the watermelon flowering stage; T1 represents the soil with wheat straw addition at the watermelon flowering stage; CK2 represents the soil without wheat straw addition at the watermelon fruiting stage; T2 represents the soil with wheat straw addition at the watermelon fruiting stage. Data represent mean ± SD, n=9. Same letters within a column indicate no significant differences between the means (*P*>0.05).

**Table S2a** Relative abundances (1>%) of bacterial phyla of all soil samples

| Phylum | **CK1** | **T1** | **CK2** | **T2** |
| --- | --- | --- | --- | --- |
| Proteobacteria | 43.25±2.18a | 42.21±2.74a | 35.04±2.58a | 35.08±1.95a |
| Actinobacteria | 8.86±0.90b | 14.61±1.30a | 14.65±1.92b | 19.23±1.16a |
| Gemmatimonadetes | 13.44±1.24a | 11.77±0.71b | 10.69±1.51a | 9.42±0.76a |
| Planctomycetes | 7.12±1.00a | 6.06±1.09a | 9.73±1.98a | 8.82±1.91a |
| Chloroflexi | 4.06±0.23b | 4.86±0.43a | 5.68±0.52b | 6.27±0.30a |
| Acidobacteria | 4.50±0.50a | 4.59±0.36a | 5.52±0.56a | 5.28±0.20a |
| Bacteroidetes | 5.15±0.28a | 5.12±0.33a | 3.54±0.39a | 3.85±0.35a |
| Parcubacteria | 4.08±0.70b | 2.13±0.41a | 2.92±0.48b | 1.68±0.32a |
| Verrucomicrobia | 2.05±1.13a | 1.96±0.76a | 2.62±1.22a | 2.97±1.06a |
| Firmicutes | 2.56±1.43a | 1.94±0.38a | 2.27±0.52a | 2.09±0.63a |
| Saccharibacteria | 0.86±0.03b | 1.36±0.16a | 1.26±0.15b | 1.53±0.10a |
| Other | 3.10±0.98a | 2.36±0.89a | 1.18±0.23a | 1.06±0.24a |
| Unclassified | 0.92±0.24a | 0.96±0.24a | 2.58±0.52a | 1.95±0.35a |

**Table S2b** Relative abundances (0.1>%) of fungal phyla of all soil samples

| Phylum | CK1 | T1 | CK2 | T2 |
| --- | --- | --- | --- | --- |
| Ascomycota | 59.54±10.40b | 80.98±2.92a | 65.93±5.39a | 50.01±5.02b |
| Basidiomycota | 20.85±8.81a | 11.47±1.89b | 22.47±6.91b | 45.26±4.79a |
| Chytridiomycota | 0.24±0.24a | 0.43±0.30a | 0.47±1.21a | 0.45±0.55a |
| Mortierellomy | 0.44±0.22a | 0.38±0.20a | 0.31±0.15a | 0.13±0.10a |
| Mucoromycota | 0.00±0.00a | 0.02±0.04a | 0.00±0.01a | 0.00±0.01a |
| Glomeromycota | 0.01±0.02a | 0.01±0.03a | 0.02±0.07a | 0.00±0.00a |
| Unclassified | 18.88±8.47a | 6.67±2.72a | 10.76±6.07a | 4.11±1.18a |

CK1 represents the soil without wheat straw addition at the watermelon flowering stage; T1 represents the soil with wheat straw addition at the watermelon flowering stage; CK2 represents the soil without wheat straw addition at the watermelon fruiting stage; T2 represents the soil with wheat straw addition at the watermelon fruiting stage. Data represent mean ± SD, n=9. Different letters within a column indicate significant differences between CK1 vs T1 and CK2 vs T2 means (*P* < 0.05).

**Table S3a** Significant differences bacterial genus in flowering stage of watermelon

| **Phylum** | **Genus** | **RA(CK1)** | **Phylum** | **Genus** | **RA(T1)** |
| --- | --- | --- | --- | --- | --- |
| Nitrospirae | Nitrospira | 0.47±0.04 | Actinobacteria | Nocardioides | 1.42±0.19 |
| Deinococcus_  Thermus | Truepera | 0.37±0.05 | Actinobacteria | Streptomyces | 0.92±0.08 |
| Proteobacteria | Polycyclovorans | 0.15±0.04 | Actinobacteria | Marmoricola | 0.57±0.07 |
| Proteobacteria | Woodsholea | 0.13±0.01 | Actinobacteria | Aeromicrobium | 0.42±0.06 |
| Proteobacteria | Acidovorax | 0.13±0.02 | Actinobacteria | Amycolatopsis | 0.33±0.05 |
| Bacteroidetes | Chryseolinea | 0.13±0.02 | Proteobacteria | Sphingobium | 0.20±0.04 |
| Firmicutes | Sporosarcina | 0.08±0.04 | Bacteroidetes | Flavobacterium | 0.16±0.02 |
| Actinobacteria | Actinomadura | 0.04±0.02 | Actinobacteria | Sporichthya | 0.14±0.02 |
|  |  |  | Actinobacteria | Ilumatobacter | 0.14±0.01 |
|  |  |  | Proteobacteria | Phaselicystis | 0.14±0.02 |
|  |  |  | Actinobacteria | Nonomuraea | 0.13±0.04 |
|  |  |  | Actinobacteria | Agromyces | 0.10±0.01 |
|  |  |  | Actinobacteria | Patulibacter | 0.08±0.01 |

Values are means ± standard deviation, addition wheat straw (T1) and without wheat straw (CK1) at watermelon flowering stage (n = 9). RA represents relative abundance

**Table S3b** Significant differences bacterial genus in fruiting stage of watermelon

| **Phylum** | **Genus** | **RA (CK2)** | **Phylum** | **Genus** | **RA (T2)** |
| --- | --- | --- | --- | --- | --- |
| Planctomycetes | SM1A02 | 0.99±0.15 | Actinobacteria | Aeromicrobium | 1.37±0.10 |
| Proteobacteria | H16 | 0.57±0.04 | Actinobacteria | Nocardioides | 1.32±0.13 |
| Proteobacteria | Polycyclovorans | 0.48±0.05 | Actinobacteria | Nonomuraea | 0.46±0.05 |
| Proteobacteria | Woodsholea | 0.26±0.03 | Proteobacteria | Pseudohongiella | 0.46±0.03 |
| Acidobacteria | PAUC26f | 0.25±0.02 | Proteobacteria | Dokdonella | 0.22±0.04 |
| Deinococcus_  Thermus | Truepera | 0.24±0.06 | Chloroflexi | Roseiflexus | 0.21±0.02 |
| Proteobacteria | Pelagibius | 0.12±0.02 | Actinobacteria | Solirubrobacter | 0.20±0.02 |
| Firmicutes | Sporosarcina | 0.08±0.03 | Bacteroidetes | Flavisolibacter | 0.16±0.02 |
| Actinobacteria | Actinomadura | 0.07±0.01 | Proteobacteria | Rhizobium | 0.16±0.01 |
| Planctomycetes | I-8 | 0.07±0.02 | Chloroflexi | Herpetosiphon | 0.15±0.03 |
| Bacteroidetes | Chryseolinea | 0.07±0.01 | Actinobacteria | Ilumatobacter | 0.13±0.01 |
| Acidobacteria | Candidatus_  Solibacter | 0.07±0.01 | Proteobacteria | Dongia | 0.13±0.02 |
| Proteobacteria | Sulfurifustis | 0.06±0.01 | Proteobacteria | Pseudoxanthomonas | 0.13±0.04 |
| Proteobacteria | Nitrosospira | 0.05±0.02 | Bacteroidetes | Flavobacterium | 0.13±0.01 |
|  |  |  | Bacteroidetes | Chitinophaga | 0.11±0.02 |
|  |  |  | Proteobacteria | Ramlibacter | 0.09±0.01 |
|  |  |  | Bacteroidetes | Dyadobacter | 0.09±0.01 |
|  |  |  | Actinobacteria | Dactylosporangium | 0.09±0.01 |
|  |  |  | Proteobacteria | Sphingobium | 0.08±0.02 |
|  |  |  | Proteobacteria | Hyphomicrobium | 0.08±0.01 |
|  |  |  | Proteobacteria | Parablastomonas | 0.06±0.00 |
|  |  |  | Bacteroidetes | Fluviicola | 0.04±0.00 |
|  |  |  | Proteobacteria | brachysporum_group | 0.04±0.00 |

Values are means ± standard deviation, addition wheat straw (T2) and without wheat straw (CK2) at watermelon fruiting stage (n = 9). RA represents relative abundance

**Table S4a** Significant differences fungal genus in flowering stage of watermelon

| **Phylum** | **genus** | **RA (CK1)** | **Phylum** | **genus** | **RA (T1)** |
| --- | --- | --- | --- | --- | --- |
| Basidiomycota | Conocybe | 18.88±8.33 | Ascomycota | Schizothecium | 8.34±2.06 |
| Ascomycota | Fusarium | 9.70±5.54 | Basidiomycota | Entoloma | 3.05±1.23 |
| Ascomycota | Scopulariopsis | 4.82±1.72 | Ascomycota | Preussia | 1.64±0.94 |
| Basidiomycota | Thanatephorus | 0.24±0.18 | Ascomycota | Bipolaris | 0.31±0.35 |
|  |  |  | Ascomycota | Lecanicillium | 0.10±0.12 |

Values are means ± standard deviation, addition wheat straw (T1) and without wheat straw (CK1) at watermelon flowering stage (n = 9). RA represents relative abundance

**Table S4b** Significant differences fungal genus in fruiting stage of watermelon

| **Phylum** | **genus** | **RA (CK2)** | **Phylum** | **genus** | **RA (T2)** |
| --- | --- | --- | --- | --- | --- |
| Scopulariopsis | Scopulariopsis | 9.66±3.19 | Ascomycota | Schizothecium | 3.46±0.80 |
| Ascomycota | Cladosporium | 4.22±1.17 |  |  |  |
| Ascomycota | Aphanoascus | 3.22±1.79 |  |  |  |
| Basidiomycota | Psathyrella | 1.16±0.78 |  |  |  |
| Ascomycota | Microascus | 0.80±0.30 |  |  |  |
| Basidiomycota | Filobasidium | 0.11±0.11 |  |  |  |

Values are means ± standard deviation, addition wheat straw (T1) and without wheat straw (CK1) at watermelon fruiting stage (n = 9). RA represents relative abundance.

**Table S6** Major topological properties of the empirical phylogenetic Molecular Ecological Networks (pMENs) of bacterial communities for soil with (T1 and T2) and without (CK1 and CK2) addition wheat straw and their associated random pMENs.

|  | **Empirical networks** | | | | | | | | | **Random networks** ^d^ | | |
| --- | --- | --- | --- | --- | --- | --- | --- | --- | --- | --- | --- | --- |
|  | Soil traits | NO. of original OTUs^a^ | Similarity threshold (S­t) | Network size (n)^b^ | R^2^ of Power law | Avg connect (avg K) | Avg path length  (GD)^c^ | Avg clustering coefficient (avg CC) | Modularity (NO.of modules) | Avg path distance (GD) | Avg cluster coefficient (avg CC) | Modularity (M) |
| Flowering stage | CK1 | 300 | 0.87 | 221 | 0.808 | 7.493 | 3.557 | 0.358 | 0.440(22) | 2.977±0.044 | 0.087±0.008 | 0.295±0.006 |
|  | T1 | 300 | 0.88 | 224 | 0.715 | 7.482 | 4.273 | 0.268 | 0.403(27) | 2.969 ±0.043 | 0.152±0.013 | 0.275±0.006 |
| Fruiting stage | CK2 | 300 | 0.88 | 201 | 0.818 | 3.562 | 7.464 | 0.358 | 0.756(23) | 4.099 ±0.079 | 0.023±0.008 | 0.529±0.009 |
|  | T2 | 300 | 0.85 | 228 | 0.716 | 4.263 | 5.271 | 0.268 | 0.403(21) | 3.759 ±0.052 | 0.027±0.007 | 0.467±0.008 |

a:The number of OTUs that were originally used for network construction using the random matrix theory (RMT)-based approach.

b:The number of OTUs (i.e., nodes) in the network.

c:GD, geodesic distance.

d:The random networks were generated by rewiring all of the links of a pMEN with the identical numbers of nodes and links to the corresponding empirical pMEN.

**Table S7** The raw sequencing reads for per sample

| Sample Name | Raw Reads | Sample Name | Raw Reads |
| --- | --- | --- | --- |
| CK1-1 | 105564 | CK2-1 | 100749 |
| CK1-2 | 82407 | CK2-2 | 88913 |
| CK1-3 | 111610 | CK2-3 | 87121 |
| CK1-4 | 105817 | CK2-4 | 109838 |
| CK1-5 | 103240 | CK2-5 | 106372 |
| CK1-6 | 104512 | CK2-6 | 94127 |
| CK1-7 | 117893 | CK2-7 | 94380 |
| CK1-8 | 112589 | CK2-8 | 107688 |
| CK1-9 | 116774 | CK2-9 | 104960 |
| T1-1 | 105415 | T2-1 | 114749 |
| T1-2 | 118410 | T2-2 | 96127 |
| T1-3 | 105053 | T2-3 | 83782 |
| T1-4 | 99436 | T2-4 | 100225 |
| T1-5 | 101528 | T2-5 | 106439 |
| T1-6 | 114384 | T2-6 | 100987 |
| T1-7 | 96201 | T2-7 | 94727 |
| T1-8 | 117338 | T2-8 | 100059 |
| T1-9 | 95932 | T2-9 | 107476 |
